# Genome and low-iron response of an oceanic diatom adapted to chronic iron limitation

**DOI:** 10.1186/gb-2012-13-7-r66

**Published:** 2012-07-26

**Authors:** Markus Lommer, Michael Specht, Alexandra-Sophie Roy, Lars Kraemer, Reidar Andreson, Magdalena A Gutowska, Juliane Wolf, Sonja V Bergner, Markus B Schilhabel, Ulrich C Klostermeier, Robert G Beiko, Philip Rosenstiel, Michael Hippler, Julie LaRoche

**Affiliations:** 1 RD2 Marine Biogeochemistry, Helmholtz Centre for Ocean Research Kiel (GEOMAR), Düsternbrooker Weg 20, Kiel, D-24105, Germany; 2 Institute of Plant Biology and Biotechnology, University of Münster, Hindenburgplatz 55, Münster, D-48143, Germany; 3 Institute of Clinical Molecular Biology ICMB, Christian-Albrechts-University Kiel, Schittenhelmstrasse 12, Kiel, D-24105, Germany; 4 Department of Biology, University of Bergen, Thormøhlensgt. 53 A/B, Bergen, NO-5020, Norway; 5 Estonian Biocentre, University of Tartu, Riia 23b, Tartu, EE-51010, Estonia; 6 Institute of Physiology, Christian-Albrechts-University Kiel, Hermann-Rodewald-Strasse 5, Kiel, D-24118, Germany; 7 Faculty of Computer Science, Dalhousie University, 6050 University Avenue, Halifax, NS B3H 1W5, Canada; 8 Department of Biology, Dalhousie University, 1355 Oxford Street, Halifax, NS B3H 4J1, Canada

## Abstract

**Background:**

Biogeochemical elemental cycling is driven by primary production of biomass via phototrophic phytoplankton growth, with 40% of marine productivity being assigned to diatoms. Phytoplankton growth is widely limited by the availability of iron, an essential component of the photosynthetic apparatus. The oceanic diatom *Thalassiosira oceanica *shows a remarkable tolerance to low-iron conditions and was chosen as a model for deciphering the cellular response upon shortage of this essential micronutrient.

**Results:**

The combined efforts in genomics, transcriptomics and proteomics reveal an unexpected metabolic flexibility in response to iron availability for *T. oceanica *CCMP1005. The complex response comprises cellular retrenchment as well as remodeling of bioenergetic pathways, where the abundance of iron-rich photosynthetic proteins is lowered, whereas iron-rich mitochondrial proteins are preserved. As a consequence of iron deprivation, the photosynthetic machinery undergoes a remodeling to adjust the light energy utilization with the overall decrease in photosynthetic electron transfer complexes.

**Conclusions:**

Beneficial adaptations to low-iron environments include strategies to lower the cellular iron requirements and to enhance iron uptake. A novel contribution enhancing iron economy of phototrophic growth is observed with the iron-regulated substitution of three metal-containing fructose-bisphosphate aldolases involved in metabolic conversion of carbohydrates for enzymes that do not contain metals. Further, our data identify candidate components of a high-affinity iron-uptake system, with several of the involved genes and domains originating from duplication events. A high genomic plasticity, as seen from the fraction of genes acquired through horizontal gene transfer, provides the platform for these complex adaptations to a low-iron world.

## Background

Diatoms are important primary producers in the ocean [1], contributing approximately 40% to global marine productivity. Although diatoms often dominate phytoplankton communities in nutrient-rich ecosystems, members of this diverse group are also adapted to survive and persist in nutrient-limited conditions. The development of large diatom blooms upon nutrient resupply demonstrates the metabolic plasticity inherent to their ability to recover rapidly from nutrient limitation.

Iron is an essential nutrient for all organisms and in particular for photoautotrophic organisms. It functions as a powerful electron carrier in iron-sulfur- and heme-containing proteins and as such is a required component of the photosynthetic apparatus. Solubility of iron in seawater is low and phytoplankton growth in marine habitats is often limited by iron availability. This is best illustrated in high-nitrate low-chlorophyll (HNLC) regions, remote oceanic areas that lack any form of regular iron supply and suffer from a persistent shortage of this micronutrient. Here, although other commonly limiting nutrients like nitrate or phosphate are present at high concentrations, primary productivity - and biomass as a whole - is low [2].

Numerous large-scale iron fertilization experiments have confirmed that iron is the limiting nutrient in HNLC regions [3]. Phytoplankton blooms induced by iron fertilization were dominated by diatoms and carbon export to the deep-sea floor could be observed in some cases. The strong response of diatoms to the input of iron in HNLC regions has been a motivation for exploring large-scale iron fertilization as a possible bioengineering strategy to sequester CO_2 _into the ocean in HNLC regions, which are otherwise rich in macronutrients.

Genome projects on the model organisms *Thalassiosira pseudonana *[4] and *Phaeodactylum tricornutum *[5] have already generated a wealth of insights into the metabolic complexity of diatoms [6], a consequence of the secondary endosymbiosis event that gave rise to this group [7]. This secondary endosymbiosis brought together the benefits of a heterotrophic host and the 'red'-type photosynthesis of red alga cells, which already have an elemental composition low in iron [8].

The impact of iron availability on phytoplankton growth has led to the evolution of strategies to counteract iron limitation. Well established parts of the low-iron response found in diverse phytoplankton species are the reduction of the chloroplast system, the corresponding development of a chlorotic phenotype, compensation mechanisms (replacement of iron-rich elements with iron-poor substitutes) and the activation of high-affinity iron-uptake systems [9]. The substitution of ferredoxin by flavodoxin [10], the use of plastocyanin instead of cytochrome c_6 _[11] and a variant stoichiometry of photosynthetic complexes [12] are notable adaptive strategies to facilitate diatom growth in low-iron conditions.

Oceanic and neritic phytoplankton species can be distinguished from each other by their growth characteristics and their tolerance to nutrient limitation [13]. Unlike many other *Thalassiosira *species that are predominantly found in coastal waters, *Thalassiosira oceanica *is adapted to oligotrophic conditions and is highly tolerant to iron limitation in particular. Therefore, we chose *T. oceanica *CCMP1005 as a model for a comprehensive analysis of its low-iron response in the context of genomic information.

Here, we explore the complex cellular response of *T. oceanica *to low-iron conditions with genomics, transcriptomics and proteomics approaches complemented by reverse transcription-quantitative PCR (RT-qPCR) analyses. We present a metabolic reconstruction of the iron limitation response based on the transcriptomics data from cells grown under iron-limited versus iron-replete conditions. A metabolic isotope labeling approach using ^14^N/^15^N was established for *T. oceanica *and showed the response to iron limitation at the protein expression level in a marine diatom for the first time. General characteristics of the 'diatom' low-iron response and its ecological implications are discussed, as well as the constraints for species-specific adaptations to low-iron environments.

## Results

### Characteristics of the *T. oceanica *genome

**Figure 1:**
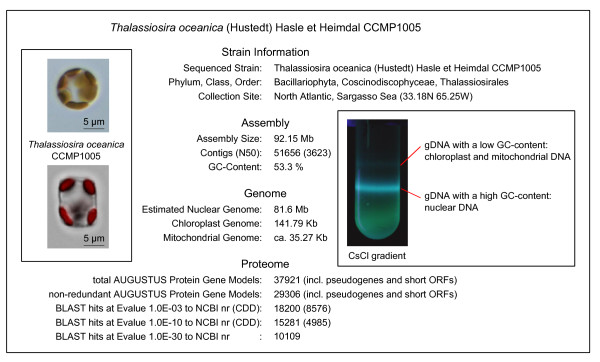
***T. oceanica *CCMP1005 genome statistics**. The sequenced strain *T. oceanica *CCMP1005 belongs to the Centrales group of radially symmetric diatoms and was first isolated from the oligotrophic Sargasso Sea by R Guillard. At 92.15 Mb, our genome assembly is slightly larger than the expected haploid genome size of 81.6 Mb, suggesting some redundancy in the current assembly. The genuine AUGUSTUS gene model predictions include a large fraction of pseudogenes and short ORFs that show no homology to any proteins from the NCBI nr database at a reasonable E-value cutoff. Left inset contains light microscopy images of the sequenced organism in valve view (upper image, chloroplasts brown) and girdle view (lower image, chloroplasts red from overlay of chlorophyll autofluorescence). Right inset shows the separation of nuclear and organellar DNA in a CsCl density gradient. Stained DNA emits blue fluorescence upon excitation with UV light.

The genome of the centric diatom *T. oceanica *CCMP1005 (Figure 1) was *de novo *assembled from 725 Mb of Roche 454 sequence read information, generated using nuclear genomic DNA (gDNA) of an axenic clonal culture as substrate [14]. The current assembly version comprises 51,656 contigs of total size 92.15 Mb at N50 = 3,623 (that is, 50% of the genomic sequence information is present as contigs ≥3,623 bases). From a median 8.7-fold coverage of long contigs (≥10 kb) we estimated a true haploid nuclear genome size of 81.6 Mb, suggesting some redundancy in the current assembly. This estimate is in good agreement with the 159 Mb measured by van Dassow *et al*. [15] for the diploid G1 DNA content. The gene finder tool AUGUSTUS [16] predicts 37,921 protein gene models that cluster into a non-redundant set of 29,306 models including pseudogenes and short ORFs; 10,109 models have BLAST hits to the National Center for Biotechnology Information (NCBI) nr protein database at a conservative E-value cutoff of 1.0E-30 and thus are more indicative of the expected true protein-coding gene number (that is, expressed genes excluding pseudogenes and short ORFs). Best BLAST hits are listed in Additional file 1. In Table 1 we present an overview of the most abundant Clusters of Orthologous Groups (COG) domains in *T. oceanica*. The abundances of diverse groups of ATPases were overall very similar to those for other diatoms. A group of 19 chitinases is shared between the two centric *Thalassiosira *species.

**Table 1:** Most abundant protein domains in diatom genomes

COG		To	Tp	Pt	Fc
ATPases					
COG0515	SPS1, serine/threonine protein kinase	115	132	119	137
COG0464	SpoVK, ATPases of the AAA+ class	90	43	38	44
COG1132	MdlB, ABC-type multidrug transport system, ATPase and permease components	54	44	47	51
COG1222	RPT1, ATP-dependent 26S proteasome regulatory subunit	50	41	37	42
COG0465	HflB, ATP-dependent Zn proteases	49	37	35	39
COG1223	Predicted ATPase (AAA+ superfamily)	44	39	34	41
COG3899	Predicted ATPase	42	48	11	2
COG2274	SunT, ABC-type bacteriocin/lantibiotic exporters, contain an amino-terminal double-glycine peptidase domain	42	52	50	61
COG5265	ATM1, ABC-type transport system involved in Fe-S cluster assembly, permease and ATPase components	40	33	30	33
COG4618	ArpD, ABC-type protease/lipase transport system, ATPase and permease components	39	41	42	43
COG4987	CydC, ABC-type transport system involved in cytochrome bd biosynthesis, fused ATPase and permease components	31	50	46	56
COG4988	CydD, ABC-type transport system involved in cytochrome bd biosynthesis, ATPase and permease components	29	52	49	60
COG0488	Uup, ATPase components of ABC transporters with duplicated ATPase domains	29	50	46	53
COG1131	CcmA, ABC-type multidrug transport system, ATPase component	22	56	52	65
COG0661	AarF, predicted unusual protein kinase	21	21	22	26
COG0474	MgtA, cation transport ATPase	12	19	19	18
					
Basic cellular functions					
COG0513	SrmB, superfamily II DNA and RNA helicases	46	48	44	54
COG0553	HepA, superfamily II DNA/RNA helicases, SNF2 family	35	27	24	36
COG5059	KIP1, kinesin-like protein	24	25	15	14
COG1643	HrpA, HrpA-like helicases	21	14	9	20
COG0443	DnaK, molecular chaperone	18	14	9	10
COG5021	HUL4, ubiquitin-protein ligase	15	7	8	8
COG5022	Myosin heavy chain	14	11	9	9
COG1249	Lpd, Pyruvate/2-oxoglutarate dehydrogenase complex, dihydro-lipoamide dehydrogenase (E3) component, and related enzymes	12	14	13	20
					
Chitinases					
COG3325	ChiA, chitinase	19	19	1	0

Protein domains are listed based on Clusters of Orthologous Groups COG with an E-value threshold of 1.0E-10. To, *Thalassiosira oceanica*; Tp, *Thalassiosira pseudonana*; Pt, *Phaeodactylum tricornutum*; Fc. *Fragilariopsis cylindrus*.

The chloroplast genome has been published previously [17]. The mitochondrial genome encodes 31 protein genes and is represented by two contigs at a total of 35.3 kb (excluding the characteristic mitochondrial repeats). The current genome assembly, AUGUSTUS protein gene models, ESTs and proteomics peptides as well as updated versions thereof are publicly accessible with the *Thalassiosira oceanica *Genome Browser [18].

With an estimated haploid size of approximately 80 Mb, the genome of *T. oceanica *is significantly larger than those of *T. pseudonana *(approximately 34 Mb) or *P. tricornutum *(approximately 28 Mb), and rather comparable to that of *Fragilariopsis cylindrus *(approximately 80 Mb) [19]. The genome expansion has occurred by DNA recruitment from both internal and external DNA sources.

**Figure 2:**
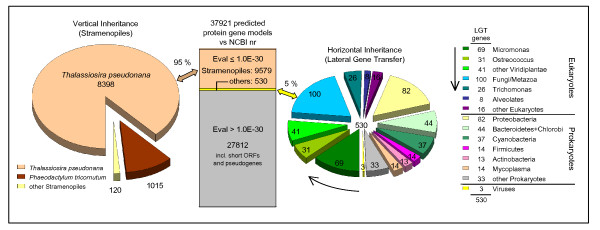
**Vertical versus horizontal inheritance of genes**. For evaluation of the extent of laterally acquired genes we focused on the 10,109 AUGUSTUS gene models that have homologs in the NCBI nr protein database at a conservative E-value cutoff of 1.0E-30 (middle bar). A significant fraction of the vertically inherited genes (left) is not shared with the closest relative *T. pseudonana*, but rather with *P. tricornutum*. Genes acquired through mechanisms of lateral gene transfer (LGT; right) appear to be derived from diverse prokaryotic and eukaryotic taxons with the highest contribution by the green algal genus *Micromonas*.

A best BLAST hit analysis indicated a putative vertical inheritance for greater than 95% of the 10,109 predicted genes (that is, any genes that have not been acquired by a horizontal transfer event), with most of the genes (88%) having a match in the genome of *T. pseudonana*, the most closely related species for which a sequenced genome is available (Figure 2). However, a significant fraction (10%) of the genes mapped to *P. tricornutum *instead. This could have resulted from frequent gene loss/replacement events in the genome of *T. pseudonana*, thereby reflecting the overall high capacity for horizontal gene transfer in diatoms [5]. Alternatively, the small genome size of *T. pseudonana *may have arisen from reductional trends in this species.

Further, the best BLAST screening revealed 530 genes whose best hits are assigned to taxa of diverse sources, indicating a putative lateral acquisition for these genes. The taxonomic distribution of the best BLAST hits at a conservative E-value cutoff of 1.0E-30 is presented in Figure 2. More refined phylogenetic analyses were obtained for 198 of these 530 genes (Additional file 2). Of the 198 cases we examined, 180 had sister groups that contained no stramenopiles. The sister groups for the remaining 18 cases contained a heterogenous mix of taxa, suggesting frequent transfer between taxa for the respective genes (Figure S1 in Additional file 3). Accordingly, a minimum of 1.8% of the 10,109 AUGUSTUS genes were confirmed to be from lateral gene transfer (LGT) based on the phylogenetic analyses. However, this may rise to 5% as more sequence information becomes available for the remaining >300 genes for which the limited number of homologous sequences did not permit construction of phylogenetic trees. The group of genes for which phylogenetic trees are available indicated that genes from LGT could be assigned as prokaryotic (35%) and eukaryotic (59%) with approximately 10% of questionable taxonomic assignments. Among the eukaryotic taxa are several expected to be present in the ecological niche of *T. oceanica*, like the green algal genuses *Micromonas *and *Ostreococcus*.

Genomic expansion originating from internal DNA sources may happen from genomic duplication events or transposon activity. In *T. oceanica *we observe several paralogous gene pairs that could be the result of either mechanism (Figure S2 in Additional file 3). Notably, several iron-regulated genes have either been duplicated (for example, the *ISIP1 *genes *ISIP1A *and *ISIP1B *and the flavodoxin genes *FLDA1 *and *FLDA2*) or contain domain duplications (for example, *CREGx2*) as discussed below.

### Physiology of the low-iron response: Fe(-) versus Fe(+)

The variable to maximal fluorescence ratio F_v_/F_m_, an indicator of Fe-limitation in the laboratory [20], was used as a rapid measure of the physiological status in Fe-replete and Fe-limited cultures of *T. oceanica *harvested in late exponential growth phase. The growth rate of iron-limited cells in exponential phase was accordingly much smaller than for iron-replete cells (Table 2), and cellular protein content was 50% lower.

**Table 2:** Physiology of the *T. oceanica *low-iron response

Fe(+)		Fe(-)
0.5 - 0.6	F_v_/F_m_	0.2 - 0.3
0.73 ± 0.01	Growth rate (µ)(day^-1^)	0.28 ± 0.02
4	Chloroplasts/cell	2
409 ± 48	Chlorophyll a/cell (fg)	58 ± 27
122 ± 3	Cell surface area (µm^2^)	140 ± 5
100 ± 4	Cell volume (µm^3^)	80 ± 5
15.3 ± 0.9	Single chloroplast surface area (µm^2^)	12.0 ± 0.8
4.7 ± 0.3	Single chloroplast volume (µm^3^)	3.5 ± 0.2

Cellular dimensions and physiological parameters are compared between nutrient replete Fe(+) and iron-limited Fe(-) cells of exponentially growing *T. oceanica *cultures.

**Figure 3:**
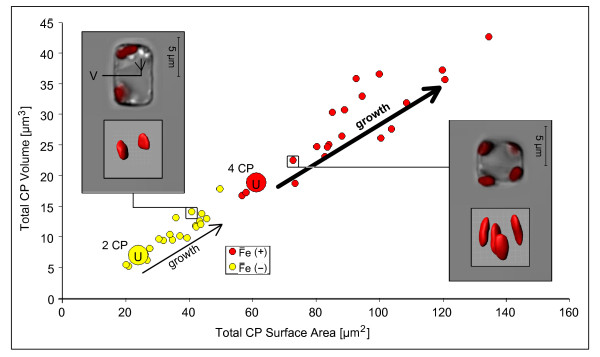
**Reduction of the chloroplast system**. The approximate dimensions of the photosynthetic machinery were assessed using confocal laser scanning microscopy and subsequent three-dimensional reconstruction of the chlorophyll autofluorescence signal. A plot of total cellular chloroplast volume versus total cellular chloroplast surface area shows a reduction of the chloroplast system in iron-limited *T. oceanica *cells. Iron-limited cells have a reduced number of two chloroplasts instead of four. Total chloroplast dimensions for individual cells (small circles) are distributed over a range spanning the two-fold increase in volume and surface that accompanies chloroplast duplication during cellular growth. Inserts show an overlay transmission and chlorophyll autofluorescence image (top) and the respective three-dimensional chloroplast reconstruction (bottom). The left insert illustrates an iron-limited cell close to dividing with two nearly duplicated chloroplasts. Note the characteristic increase in vesiculation of the cellular interior at low-iron. An iron-replete cell at the beginning of its cell cycle (shortly after division) contains four chloroplasts (right insert). CP, chloroplast; U, cell at the beginning of its cell cycle ('unit cell'); V, vesicle.

The cell volume of iron-limited *T. oceanica *was smaller than that of iron-replete cells (Table 2), whereas the cells had a larger surface area to volume ratio at low-iron due to a smaller diameter (4.7 ± 0.1 versus 5.9 ± 0.1 µm) and a larger length (7.0 ± 0.4 versus 5.5 ± 0.2 µm). This imposed an elongated phenotype on the cells, but at the same time increased the surface/volume ratio by 43% (1.75 versus 1.22). The increase in surface/volume ratio is expected to favor the uptake of nutrients (that is, iron) into the cell [21]. The intracellular space of iron-limited cells exhibited increased vesiculation (Figure 3).

Under low-iron conditions *T. oceanica *cells show a severe decrease in chlorophyll content (Table 2). This chlorosis response of iron-limited *T. oceanica *is further accompanied by a decrease in cellular chloroplast volume and in total cellular chloroplast surface area (Figure 3). Iron-limited cells have reduced the number of chloroplasts to two instead of four in the iron-replete counterpart, and these are also smaller in size. Total chloroplast dimensions for individual cells were distributed over a range spanning the two-fold increase in volume and surface, thereby reflecting chloroplast duplication during cellular growth.

### Transcriptomics

**Figure 4:**
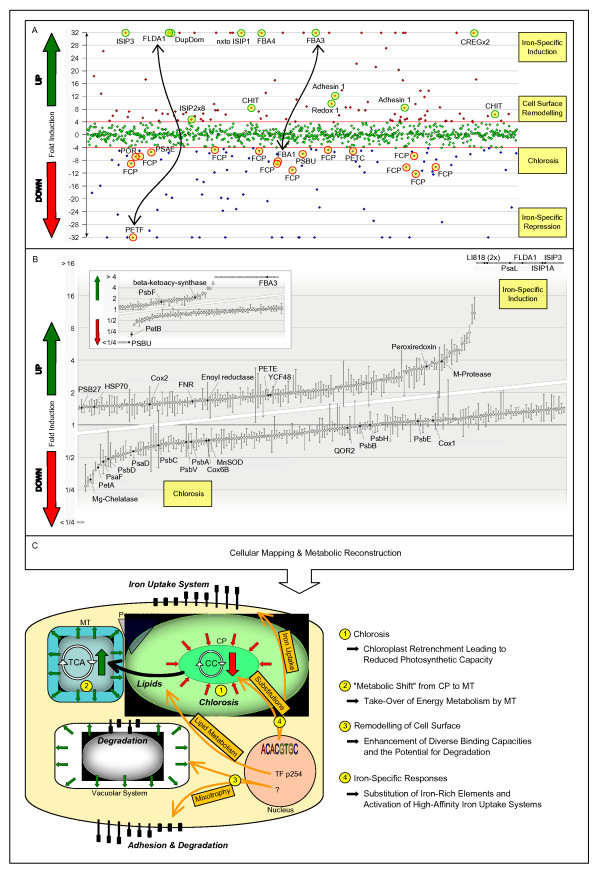
**Basic cellular changes at low-iron**. Differential gene expression of exponentially growing iron-limited versus iron-replete *T. oceanica *cells was assessed from global transcriptomics and proteomics approaches. **(a) **Transcriptomics data were screened with T-ACE, a transcriptome database browser that plots the assembled transcript fragments according to their differential regulation as inferred from differential read contribution of Fe(-) and Fe(+) libraries to each transcript contig. **(b) **For the proteomics data the differential regulation of each gene product is represented by the median of all PBC (peptide/SDS-PAGE band/charge) ratios assigned to it, with error bars constructed from the first and third quartiles. The main plot shows proteins with at least two PBC values, inset contains proteins with a single PBC value. **(c) **Only a subset of low-iron responsive genes could be assigned a robust annotation and were suitable for mapping to a cellular scheme. Accordingly, the cellular response of *T. oceanica *to low-iron was inferred from the mapping of a representative selection of genes (see text) and their respective differential regulation on the transcript and protein levels. The most pronounced elements of the complex response are chloroplast retrenchment (chlorosis) and the consequential take-over of energy metabolism by the mitochondrial system (metabolic shift). Diverse surface-related binding capacities and the potential for degrading organic matter are enhanced, suggesting a putative mixotrophic response (mixotrophy). The strongest transcriptional response is seen from genes involved in iron-uptake or compensational substitutions (4). This iron-specific part of the cellular response may be mediated by a conserved promoter motif identified in this work. CC, Calvin-Benson-Bassham cycle; CP, chloroplast; MT, mitochondria; TCA, tricarboxylic acid cycle; TF, transcription factor.

For an in-depth analysis of the *T. oceanica *low-iron response, we focused on approximately 300 genes that were identified from a log-likelihood ratio test statistic [22] as significantly differentially regulated and that could be assigned a specific function (Figure 4a). Some additional genes for paralogous proteins were added. These were selected on the basis of their involvement in substitution between related proteins under iron-limited and replete conditions, or as members of a protein family exhibiting a differential response to low-iron conditions. In such cases, the response of a specific gene is better understood in the context of its respective group or family. The complement of organellar genes (encoded by the chloroplast and mitochondrial genome) was added as representative for the two well-defined and important pathways of photosynthetic and respiratory electron transport, or as proxy for organellar activity, respectively. A list of abbreviations for the genes discussed in this work can be found in Additional file 4. All sequences of the selected proteins are provided in Additional file 5, and the corresponding annotation is provided in Additional file 6.

To determine the major metabolic differences found in iron-limited compared to iron-replete growth conditions, all annotated gene products together with their respective expression data were mapped on a cellular scheme. The major cellular trends that could be deduced are summarized in Figure 4c. Identifier and detailed information on the discussed proteins (Additional file 7) are given in Additional file 8. In the following, proteins are referred to as exemplary (HSF1, p271) with HSF1 reflecting the gene name (or shortcut) and p271 being the identifier of its respective manually improved protein model (Additional file 5).

Under stress conditions, maintaining cellular integrity is crucial to survival. During iron limitation, the electron flow through the impaired photosynthetic machinery leads to enhanced production of reactive oxygen species that damage biomolecules located near the thylakoid membranes [23]. The need for protein repair and refolding induces an 'oxidative stress response' that is presumably coordinated by up-regulated heat shock factors (HSF1, p271; HSF2, p256). While all other chloroplast-encoded transcripts were down-regulated in the course of the general chlorosis response, the chloroplast chaperones dnaK and clpC were up-regulated. Additionally, an LHCSR (light harvesting complex stress responsive subunit) ortholog (LI818, p170), belonging to the FCP (fucoxanthin-chlorophyll a/c-binding protein) family of light-harvesting proteins and implicated in efficient non-photochemical quenching [24,25], showed an increased transcript level.

The development of a chlorotic phenotype and the corresponding retrenchment of the chloroplast system is the most pronounced cellular response to low iron. Accordingly, we find substantial changes in organellar transcript levels, which suggests that major functions related to the cellular energy metabolism are adopted by the mitochondrial system instead ('metabolic shift'). Chloroplast transcript levels decreased (2,026 Fe(-) versus 14,931 Fe(+) total chloroplast reads), while mitochondrial transcripts showed a two-fold increase (31,261 Fe(-) versus 18,136 Fe(+) total mitochondrial reads). Much of this effect can be attributed to the organellar rRNA operons, whose transcription is indicative of organellar translational activity (Figure S3 in Additional file 3). In parallel, diverse nuclear-encoded but chloroplast-targeted gene products were down-regulated. These included genes coding for enzymes involved in chlorophyll biosynthesis and the Calvin cycle, as well as components of the light reaction, such as photosystem (PS) subunits and several FCPs. Conversely, components of the mitochondrial respiratory chain, like cytochrome c oxidase, cytochrome b and several subunits of the NADH dehydrogenase, were up-regulated. This was also seen for a mitochondrial ATP/ADP-translocase (p242) involved in the transport of energy equivalents.

Cellular retrenchment (that is, the reduction of cellular biomass and activity) and decreased growth rates are general responses of nutrient-limited cells [13]. While chloroplast reduction was readily observable in iron limitation due to the visual predominance of these organelles in the cells, we also saw indications of a general cellular retrenchment in the transcriptional response. The expression level of the 18S rRNA gene (represented by 1,154 Fe(-) versus 2,691 Fe(+) reads) suggests a lower translation rate under iron limitation. Though such inferences must be taken with care, this would be in agreement with the decreased growth rate and lower biomass, as cellular rRNA correlates with cellular biomass. The strong up-regulation of mitochondrial isocitrate lyase (ICL, p419) and glutamine synthetase (GS, p302) suggests biomass recycling strategies to avoid losing fixed carbon and nitrogen during the metabolite conversions associated with enhanced respiration. The isocitrate lyase bridges the two decarboxylation steps of the mitochondrial citric acid cycle (carried out by isocitrate dehydrogenase and α-ketoglutarate dehydrogenase), thereby preserving carbon as glyoxylate. The glutamine synthetase reincorporates free ammonium, preserving nitrogen as glutamine. Under low-iron conditions, utilization of ammonium is energetically advantageous due to the high iron requirements of the nitrate assimilation pathway [26]. The concerted action of cellular retrenchment and biomass recycling allows for prolonged growth despite reduced carbon assimilation, thereby increasing the probability of cell survival.

**Figure 5:**
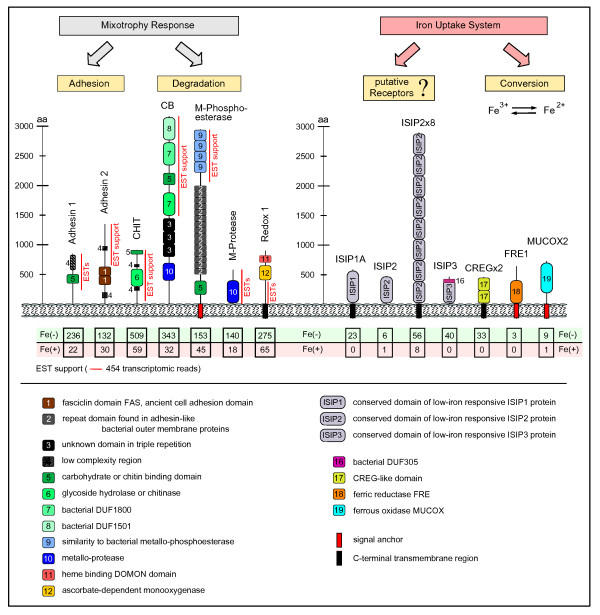
**Hypothetical categorization of low-iron-inducible cell surface proteins**. In low-iron conditions we find an up-regulation of diverse genes, whose products are targeted to the secretory pathway, suggesting extensive cell surface remodeling. Many of these are predicted to be involved in adhesion or degradation processes and might contribute to enhancing the overall cellular capacity to bind and process external organic matter. We provide a hypothetical categorization for highly responsive genes that can be assigned to this function. While some of the gene products can be placed in the context of iron uptake (right), others are less well defined, but contain a variety of conserved domains involved in adhesion or degradation of organic matter (left). Especially for larger genes, EST support is patchy, suggesting possible inaccuracies in AUGUSTUS gene modeling. Differential read contribution from the Fe(-) and Fe(+) libraries to each transcript contig (ESTs) is taken as a measure for the differential transcription of the respective gene.

Diverse genes, whose products are targeted to the secretory pathway, are up-regulated under iron limitation, suggesting extensive cell-surface remodeling as also observed for iron-limited *P. tricornutum *[20]. Many of these genes are assigned adhesive or degradative functions. An enhanced capacity for adhesion favors recruitment of organic matter to the cell. As organic matter can be a rich and complex source for various nutrients, including iron, its recruitment to the cellular surface represents a required first step in iron uptake. Besides providing a source of iron, the bound organic matter could also serve as a source for other nutrients like nitrogen or phosphorus in the context of facultative mixotrophy. Example genes assignable to such a hypothetical scenario and highly responsive to low iron are given in Figure 5 and include *Adhesin 1 *(p329), *CB *(Carbohydrate-binding 1, p230), *CHIT *(chitinase, p88), *M-Phosphoesterase *(p323), *M-Protease *(p279), *Redox 1 *(p232). However, under the photoautotrophic experimental conditions, the cultures lacked any external organic carbon source except the essential vitamins.

A straightforward strategy to survive in low-iron conditions is to lower cellular iron requirements by replacing components that are rich in iron with iron-free substitutes that are functionally equivalent, like the substitution of the chloroplast electron carrier ferredoxin with flavodoxin [10]. The genome of *T. oceanica *encodes two cytochrome c_6 _genes and one plastocyanin gene. While the cytochrome c_6 _genes *CYTC6A *and *CYTC6B *are found to be weakly expressed, the plastocyanin gene *PETE *shows high expression under high-iron conditions with a characteristic decrease in low-iron conditions as seen from many constitutively expressed chloroplast genes in the course of the chlorosis response. This suggests a constitutive use of plastocyanin (PETE, p175) instead of cytochrome c_6 _for photosynthetic electron transport and is consistent with prior findings [11,12]. Constitutive expression of plastocyanin could certainly be regarded as a specific adaptation to low-iron regimes, although the retention of the cytochrome c_6 _genes suggests that these may play a role under specific environmental conditions. Fructose-bisphosphate aldolase (FBA) genes are redundant in some diatoms and have recently been described in more detail for *P. tricornutum *[27]. The *T. oceanica *genome, too, was found to encode several FBA enzymes, with the cytosol, the chloroplast stroma and the chloroplast pyrenoid harboring two FBA enzymes each (FBA1, p380, and FBA3, p153, in the chloroplast pyrenoid; FBA2, p381, and FBA5, AUG_g19407, in the chloroplast stroma; FBA6, AUG_g24977, and FBA4, p154, in the cytosol). As is the case in *Phaeodactylum*, one of the *T. oceanica *FBAs from each compartment (FBA1, FBA2, FBA6) appears to act through metal catalysis (class II) while the second (FBA3, FBA5, FBA4) is predicted to use Schiff-base catalysis (class I) instead. While the metal cofactor of different class II FBAs was found to be Mn^2+ ^[28], Zn^2+ ^[29] or Cd^2+ ^[30] in *Escherichia coli*, the orthologous FBAs of *T. oceanica *apparently are differentially regulated through the availability of iron, suggesting the involved metal in these enzymes might be Fe^2+^, and implying a pairwise substitution by class I enzymes.

An essential part of iron-uptake systems are ferric reductases (FREs) and ferrous oxidases (MUCOX proteins) that act on the interconversion of the two ionic species Fe^3+ ^and Fe^2+^. In the iron-limited transcriptome we find an up-regulated putative ferric reductase (FRE1, p157) and an up-regulated multicopper oxidase (MUCOX2, p67) that shows characteristics of a ferrous oxidase. Their differential regulation with respect to iron availability makes them candidates for iron-specific reductase and oxidase involved in iron uptake (Figure 5). Iron uptake requires initial binding of iron and/or iron complexes. The involved receptors are presently unknown, though a number of genes, exclusively expressed under iron limitation, are targeted to the cell surface, making them candidates for iron-binding receptors. The low-iron-responsive gene *ISIP1 *(Iron-starvation induced protein) was first identified in *P. tricornutum*, but has conserved orthologs in *T. oceanica *(*ISIP1A*, p159) and *F. cylindrus*. We provide further evidence for a role of the ISIP1 protein as a putative receptor below. Additional members in this group are ISIP2 (p160) and ISIP3 (p161), both represented by orthologs in *P. tricornutum *as well. Further, we list some proteins that contain duplicated domains known from *P. tricornutum *low-iron-responsive genes, like an eight-fold duplicated ISIP2-like subdomain (ISIP2x8, p84) or a duplicated CREG-like domain (CREGx2, p90). Duplication of iron-binding domains would directly enhance the capacity for iron binding and enable increased uptake kinetics [26].

Non-ribosomal peptide synthases (NRPSs) [31] are responsible for the production of peptide antibiotics or - in some cases - siderophores that are capable of binding iron [32]. In addition to a conserved fungal NRPS (NRPS1, p174) with orthologs in *T. pseudonana *and *P. tricornutum*, we find a putatively cytosolic NRPS of bacterial origin (NRPS2, p173) up-regulated in low-iron conditions. Co-regulated with this bacterial NRPS is a multidrug resistance-associated protein (MRP, p57) that might be involved in the export of the respective peptide products. The up-regulation of NRPSs likely indicates a defense mechanism in response to enhanced competition (either for iron or, under the premise of facultative mixotrophy, for organic matter).

We observe the induction of a reverse transcriptase (RT, p222) and a CRE-like recombinase (CRE, p321), potentially indicating an activation of mobile elements under iron limitation. These enzymes might also be involved in gene and/or domain duplication events through reverse transcription and genomic integration of cellular mRNA copies. Thereby, this molecular system may provide a link between environmental stresses and the structural dynamics of the diatom genome.

### Proteomics

The transcriptomic data of *T. oceanica *unveils extensive changes in cellular transcript levels in response to iron limitation. Although informative, transcript abundances do not necessarily reflect cellular protein levels [33]. We therefore supplemented the transcriptomic data with proteomic data to determine the protein complement in action under the defined iron-replete and iron-limited growth conditions. Figure 4b illustrates the dynamic range of differential abundances for all proteins detected by liquid chromatography-tandem mass spectrometry (LC-MS/MS) relative to equal amounts of total cellular protein for both conditions. The induction of flavodoxin is a hallmark of iron-deficiency responses in many diatoms and cyanobacteria (see above). In accordance with the transcriptome response, flavodoxin as well as ISIPs or class I FBAs could only be found under iron limitation. The extent of correlation between proteomics and transcriptomics data was assessed through plotting the relative abundance data from peptides (proteomics P) against the relative abundance data from their corresponding transcripts (transcriptomics T) (Figure S4 in Additional file 3). A stretched cluster along the y-axis indicates a high dynamic range of the transcriptomics data, while the proteomics data is more uniform for this group.

Both transcriptomics and proteomics data are biased towards highly abundant transcripts/proteins. Especially the proteomics data, despite its relatively high number of signals, could resolve only a subset of the protein complement. Accordingly, we interpret the complement of differentially regulated genes and proteins recovered from both approaches as complementary in the information that they provide, and we do not expect them to show a complete overlap. However, the overlap in the response for the specifically induced proteins ISIP1 and class I FBAs shows that the data from both approaches are, in general, in good agreement with each other.

In the proteomics data it is of specific interest to have a closer look at proteins of the photosynthetic machinery. Chloroplast ribosomal proteins provide an appropriate internal reference for the regulation of chloroplast proteins and indicate a down-regulation of the ribosomes at a ratio of 0.8 relative to the iron-replete proteome. Protein subunits of PS I were reduced about two-fold under low iron conditions (0.45), except PsaL, which was only found under iron limitation. In cyanobacteria, PsaL, generally important for trimer formation, facilitates the formation of IsiA (iron stress induced protein A) rings around PS I monomers under iron-deprivation [34]. We speculate that PsaL might be involved in the organization of PS I light-harvesting structures specifically formed under low-iron conditions and/or oligomerization of PS I in iron-limited *T. oceanica*. Subunits of the iron-containing cytochrome b_6_/f (cyt b_6_/f) complex, were down-regulated, with ratios of 0.2 and 0.32. In contrast, PS II subunits PsbB, PsbC, PsbE, PsbH and PsbV remained almost constant, with ratios at about 1.1. While the PS II core complex seems to be retained to some extent, the labile D1 protein is down-regulated at 0.7, probably reflecting a proportional decrease in functional PS II. The differential regulation of the two photosystems (0.45 for PS I versus 0.7 for PS II D1 protein) supports an adaptive significance for the remodeling of the photosynthetic architecture under iron limitation, in contrast to earlier findings [12].

While PS I and cyt b_6_/f complexes were down-regulated two- to threefold, it was still possible to detect the iron-rich mitochondrial complexes under iron limitation. Relative protein quantification was possible for subunits of complex III, complex IV and the ATPase with low-to-high iron ratios ranging from 0.95 for QOR2 (a NADPH-dependent quinone oxidoreductase) to 1.7 for the beta subunit of the mitochondrial cytochrome c oxidase (Figure 4b). This is in agreement with the transcriptomic data and supports the idea that mitochondrial electron transfer protein complexes are preserved under iron limitation relative to photosynthetic electron transfer protein complexes.

While the magnesium chelatase, involved in chlorophyll synthesis, is down-regulated at 0.35, the numerous FCP light-harvesting proteins showed very diverse responses to iron limitation (Figure S5 in Additional file 3). Some FCPs showed down-regulation under low-iron whereas others were up-regulated. In particular, LHCSR-like FCPs, involved in photoprotection, were highly abundant under iron limitation, corroborating the transcriptome analysis. Notably, the xanthophyll cycle enzyme violaxanthin de-epoxidase showed significant up-regulation at 3.1, suggesting a possible linkage to the group of FCPs, which accumulate under iron limitation.

### Comparative genomics reveals extensive genomic plasticity in *T. oceanica *

We used the genome information of *T. oceanica*, *T. pseudonana*, *P. tricornutum *and *F. cylindrus *to investigate central issues of the diatom low-iron response in a comparative genomics approach.

#### Taxonomic distribution of iron-regulated genes

We screened the four diatom genomes known to date (*T. oceanica*, *T. pseudonana*, *P. tricornutum *and *F. cylindrus*) for the highly conserved iron-regulated *ISIP1*, *ISIP3*, *PETF*, *FLDA*, *CYTC6*, *PETE *and class I and II *FBA *genes (Table 3; Additional file 9). Phylogenetic trees for the important groups of flavodoxin [35] and FBA proteins are provided in Figures S6 and S7 in Additional file 3.

**Table 3:** Presence and copy number of iron-regulated genes in the genomes of ecologically distinct diatoms

Gene	Product	Destination	Mutual substitution at low-iron	Putative role in iron uptake	To	Tp	Pt	Fc
*PETF*	Ferredoxin	CP	Ferredoxin → flavodoxin (short)		1	1	1	1
*FLDA*(s)	Flavodoxin (short)	CP	Ferredoxin → flavodoxin (short)		2	0	1	1
*FLDA*(l)	Flavodoxin (long)	SP (ER?)	None (distinct functional context)		1	1	1	1
*CYTC6 *(type A)	Cytochrome c6	CP	Cytochrome c6 (type A) → plastocyanin		2	1	1	1
*CYTC6 *(type B)	Cytochrome c (?)	SP (ER?)	None (distinct functional context)		1	1	1	0
*PETE*/*PCY*	Plastocyanin	CP	Cytochrome c6 (type A) → plastocyanin		1	0	0	1
Class II *FBA *(type A)	Class II fructose-bisphosphate aldolase	CP pyrenoid (Pt FBAC1)	Class II FBA (type A) → class I FBA (type A)		1	1	1	1
Class II *FBA *(type B)	Class II fructose-bisphosphate aldolase	CP stroma (Pt FBAC2)	Class II FBA (type B) → class I FBA (type B)		1	1	1	1
Class II *FBA *(type C)	Class II fructose-bisphosphate aldolase	Cytosolic (Pt FBA3)	Class II FBA (type C) → class I FBA (type C)		1	1	1	1
Class I *FBA *(type A)	Class I fructose-bisphosphate aldolase	CP pyrenoid (Pt FBAC5)	Class II FBA (type A) → class I FBA (type A)		1	0	1	1
Class I *FBA *(type B)	Class I fructose-bisphosphate aldolase	CP stroma	Class II FBA (type B) → class I FBA (type B)		1	0	0	1
Class I *FBA *(type C)	Class I fructose-bisphosphate aldolase	Cytosolic (Pt FBA4)	Class II FBA (type C) → class I FBA (type C)		1	1	1	1
								
*ISIP1*	Iron starvation induced protein 1	Cell surface		Receptor (?)	2	0	1	3
*ISIP3*	Iron starvation induced protein 3	Cell surface		Co-receptor (?)	1	1	1	2

The coastal diatom species *T. pseudonana *(Tp) lacks several genes that are found in the genomes of diatoms with high tolerance to low-iron conditions (*T. oceanica *(To), *P. tricornutum *(Pt), *F. cylindrus *(Fc)). Listed are also the respective counterparts whose products are subject to substitution under iron-limited conditions. The conserved paralogous genes of *FLDA *and *CYTC6 *are predicted to contain a signal peptide and are assumed to act in a different functional context. CP chloroplast; ER endoplasmic reticulum; SP, secretory pathway.

The short flavodoxin isoform, plastocyanin and the class I FBAs are known or assumed to replace iron-containing counterparts under low-iron conditions. The two oceanic diatoms *T. oceanica *and *F. cylindrus*, which have some of the highest tolerance to low-iron conditions, both contain all five of the respective genes while *P. tricornutum *lacks two of them. The typical coastal species *T. pseudonana *lacks all except the gene for the cytosolic class I FBA, while at the same time having the highest requirement for iron in the group of diatoms for which genome information is currently available. Further, we find multiple copies of the *ISIP1 *gene in *T. oceanica *and *F. cylindrus*, while this gene is absent in *T. pseudonana*. The presence or copy number of these genes in the tested diatom genomes suggests an adaptive significance with respect to the low-iron conditions found in oceanic waters.

#### Domain duplications of iron-regulated cell-surface proteins

While differentially regulated genes for cell-surface proteins, identified from the low-iron response of *P. tricornutum *[20], like *ISIP1*, *ISIP2*, *FLDA *or *CREG*, represent single-copy genes encoding well-defined single-domain proteins, the situation in *T. oceanica *is different (Figure S2 in Additional file 3). Here, we find additional paralogous versions of several iron-regulated genes (*ISIP1*, *FLDA*), as well as diverse examples of domain duplications (*CREGx2*, *ISIP2x8*). In the case of iron-binding proteins the duplication of domains might provide benefits under iron limitation through a higher density of exposed domains, thereby increasing the affinity for iron at the cell surface [26].

With respect to the selective pressure encountered in the low-iron open ocean the duplication of complete genes may provide a possible mechanism for adaptation on the molecular level, in that it allows one of the two gene copies to vary, improve and optimize its iron-binding themes/motifs. This may potentially result in more efficient iron uptake. RT-qPCR allowed us to distinguish iron-regulated genes from their closely related paralogs (Figure S8 in Additional file 3).

#### Iron uptake and the cell-surface protein ISIP1

**Figure 6:**
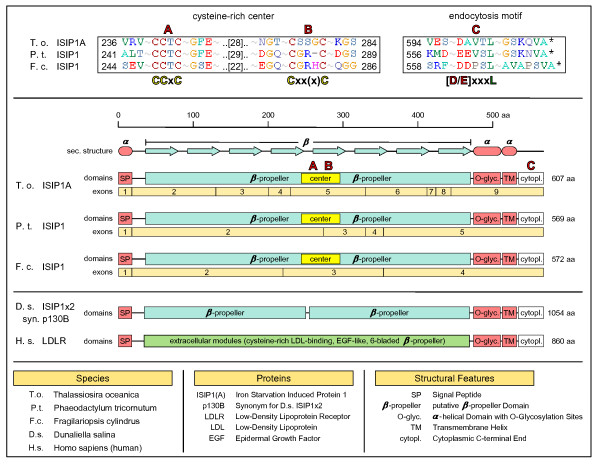
**The low-iron inducible receptor ISIP1**. ISIP1 protein models and secondary structure from *T. oceanica*, *P. tricornutum *and *F. cylindrus *are compared. Conservation between the protein orthologs is high, with identical secondary structure predictions (center). We find an amino-terminal signal peptide targeting the protein to the secretory pathway, while a carboxy-terminal transmembrane domain anchors the protein to a membrane. The major part of the protein is represented by a domain rich in β-strands that likely folds into a β-propeller-like structure. While in *D. salina *p130B (bottom) this β-propeller domain is duplicated and only distantly related to the respective diatom domains, the remainder of the protein shows a clear homology to the group of diatom ISIP1 proteins. A clue to the structure and function of ISIP1 could be the human low-density lipoprotein receptor LDLR due to its detailed characterization as a human cell-surface receptor: while its extracellular domains are very different from the single β-propeller domain of ISIP1, the remainder of the protein is again strikingly similar, which allows us to transfer the respective annotation from LDLR to the ISIP1 protein model. Accordingly, the ISIP1 protein would represent a cell-surface receptor that is anchored to the plasma membrane by a carboxy-terminal transmembrane helix. A small carboxy-terminal tail without well-defined secondary structure contains a conserved endocytosis motif C (top, right) responsible for endocytotic cycling of ISIP1. An α-helical region amino-terminal from the transmembrane helix is predicted to be O-glycosylated and thereby would serve to expose the large β-propeller as a putative receptor domain to the extracellular space. A sequence alignment of the ISIP1 proteins from *T. oceanica*, *P. tricornutum *and *F. cylindrus *illustrates that the extracellular β-propeller domain contains a cysteine-rich center, A and B (top, left). The pattern of cysteine residues is reminiscent of patterns found in Fe-S cluster proteins and might also be involved in binding Fe.

Conservation between the predicted protein orthologs of ISIP1 in *T. oceanica*, *P. tricornutum *and *F. cylindrus *was high, and the orthologs exhibited identical secondary structure predictions (Figure 6). We found an amino-terminal signal peptide targeting the protein to the secretory pathway, while a carboxy-terminal transmembrane domain anchors the protein to a membrane. The major part of the protein is represented by a domain rich in β-strands that likely folds into a β-propeller-like structure. A clue to the structure and function of ISIP1 could be the low-density lipoprotein receptor LDLR, an important cell-surface receptor in humans [36]. Although its extracellular domains differ from the single β-propeller domain of ISIP1, the remainder of the protein is strikingly similar with regard to amino acid composition and secondary structure prediction. Hence, we may transfer the respective LDLR annotation to the ISIP1 protein model.

Accordingly, the ISIP1 protein would represent a cell-surface receptor, anchored to the plasma membrane by a carboxy-terminal transmembrane helix. A small carboxy-terminal tail without well-defined secondary structure contains a conserved endocytosis motif responsible for endocytotic cycling. An α-helical region amino-terminal from the transmembrane helix is predicted to be O-glycosylated and would thereby serve to expose the large β-propeller as a putative receptor domain to the extracellular space.

An alignment of the ISIP1 proteins from *T. oceanica*, *P. tricornutum *and *F. cylindrus *illustrates that the extracellular β-propeller domain contains a cysteine-rich center (Figure 6) whose pattern is reminiscent of cysteines found in Fe-S cluster proteins and might be involved in binding Fe. The cysteine-rich center is not found in the orthologous p130B of *Dunaliella salina*, which is thought to have undergone an evolutionary change in function and interacts with transferrin-like proteins [37].

#### A conserved promoter motif associated with diverse iron-regulated genes

At the core of an organism's low-iron response are transcription factors (repressors or enhancers) and their respective binding sites (specific promoter motifs) that mediate the cellular response at the gene expression level. From pairwise promoter comparisons between the exclusively iron-regulated *ISIP1*, *FLDA *and *FBA3 *genes of *T. oceanica*, *P. tricornutum *and *F. cylindrus *using dotlet [38] and MEME [39], we identified a conserved palindromic motif 'ACACGTGC' located around position -200 from the translation start. Upon genome-wide screening, a total of 45 gene models contained the complete motif (perfect match) at a position of 150 to 250 bases before the translation start. Functional assignments for genes with positive matches were rarely possible (mostly hypothetical genes of unknown function and without significant regulation). However, the accumulation of low-iron responsive genes (*ISIP1 *and three *FBA *genes) in this group is remarkable. In Figure S9 in Additional file 3 we present only those genes whose orthologs in other species carry the motif in their promoters.

The complexity of the identified motif (A_2_T_1_C_3_G_2_) is high; its palindromic structure suggests binding of a homo- or heterodimeric protein factor. The remarkable conservation of this motif and its position (-200) relative to the translation start across three diatom species reinforces the suggestion that this motif plays a prominent role in iron-dependent gene regulation.

## Discussion

### The cellular response to iron limitation

As the most prominent part of the complex low-iron response of *T. oceanica *we observe clear indications for an extensive cellular retrenchment, best seen in the reduced number and size of the chloroplasts. The proteomics and qPCR results indicate that not only is the iron-rich photosynthetic machinery affected, but that other cellular components also encounter a large-scale reduction, resulting in decreased growth rates. In addition, low-iron cells have a significantly lower protein biomass at roughly half that of iron-replete cells. We speculate that during the transformation from a high-iron/high-biomass cell to a low-iron/low-biomass cell the cellular biomass itself may serve as a supplementary energy source to compensate for the decrease in photosynthetic performance, that is, carbon fixation and generation of ATP. This is in line with the observation of increased cellular vesiculation in low-iron conditions (light microscopy) and increased lipid metabolism (transcriptomics and proteomics). It is also consistent with the relative increase of the mitochondrial respiratory machinery as deduced from the transcriptomics and proteomics data. As phototrophy, and thereby chloroplast energy metabolism, is largely impaired in low-iron conditions, mitochondrial respiration may provide a more constant and robust energy source that is retained, that is, excluded from the observed cellular retrenchment. Hence, we can describe the principal cellular changes observed at iron limitation as a metabolic shift with a gradual take-over of the energy metabolism by the mitochondrial system. Cellular maintenance under iron-limited conditions is further supported from biomass recycling through the action of isocitrate lyase and glutamine synthetase.

Moreover, changes in the photosynthetic machinery are likely a consequence of a coordinated remodeling process, indicating an intricate regulatory network that adjusts cellular energy demand in response to the availability of iron. The active remodeling as a response to low iron is an unexpected result since it was proposed earlier that photosynthesis of *T. oceanica *is constitutively adapted to a low-iron environment [12]. The observation of distinct cellular phenotypes in iron-limited versus iron-replete cultures may be due to the differing iron levels used in iron-replete control cultures, with approximately 60 nM applied by Strzepeck and Harrison [12] compared to the saturating 10 µM FeCl_2 _used in this work, though not expected to ever occur under natural conditions. Nevertheless, our data demonstrate that *T. oceanica *possesses the potential to remodel its bioenergetic pathways in response to iron availability.

While the cultures in the present experimental setup were held under axenic photoautotrophic conditions, the situation in the natural context of the open ocean is very different. A major difference found between the artificially induced iron limitation of photoautotrophically growing cultures and the iron limitation encountered by diatoms in their natural habitats is the ubiquitous presence of particulate and dissolved organic matter in the latter, albeit dilute as in the case of the oligotrophic Sargasso Sea [40].

Some of the strongest up-regulated transcripts under low-iron conditions were found to be targeted to the secretory pathway, that is, with the cell surface or the vacuolar system as their destination. The functional annotation of the respective protein models reveals a complex suite of molecules capable of adhesion or degradative functions, suggesting a possible role in a mixotrophic context (Figure 5). Observations of diatom mixotrophy have been reported for five decades [41]. This characteristic has recently been explored for biotechnological application [42]. A principle metabolic competency for heterotrophic nutrition was demonstrated in transgenic *P. tricornutum *that were able to grow on sugar upon expression of a transgenic sugar transporter [43].

A hypothetical capability for mixotrophic nutrition and the conjoined ability to feed on dissolved and/or particulate organic matter would place diatoms in close proximity to the microbial loop responsible for recycling organic matter in the marine food web. Moreover, this would contribute to resolving the paradox of diatom survival in a low-iron world, as particulate and dissolved organic matter can be expected to be a relevant iron source. On the other hand, utilization of organic carbon/iron sources in low-iron conditions will immediately put the cell into competition with the bacterial community of diverse and specialized heterotrophs. In line with this scenario we find an up-regulation of both identified NRPS enzymes (Figure S8 in Additional file 3) that may mediate a bacterial defense response under iron-limited conditions.

### Evolutionary roots and the impact of genome plasticity on adaptation to low iron

Diatoms are the outcome of an endosymbiotic event that brought together two nutritional modes, each somewhat pre-adapted to low-iron conditions: phototrophy was contributed by the red-type plastids of the red algal endosymbiont, whose elemental composition shows a reduced iron requirement relative to the green-type plastids [8]; the host (related to ancient oomycetes) contributed an efficient heterotrophic machinery that might be retained to some extent in today's diatoms (compare [44]), thereby enabling exploitation of particulate and dissolved organic matter as a supplementary carbon and iron source. Survival under iron-limited conditions would have benefited from both the ancestral host's and symbiont's characteristics.

Additional adaptive strategies to the low-iron environment have probably also originated by means of horizontal gene transfer, with possibly as much as 5% of the conserved genes encoded in the *T. oceanica *genome being assigned to diverse taxonomic groups. This is consistent with the findings from the *Phaeodactylum *genome project [5] and appears to be a recurrent topic in diatom genomics. A prerequisite for integration of foreign DNA into a genome is its uptake, and an explanation for how lateral gene transfer might occur in heterotrophic cells has been proposed [45]. Accordingly, heterotrophic organisms would continuously take up and integrate new genes along with the organic matter they feed on, thereby creating some genetic redundancy that eventually leads to the permanent replacement of the genuine counterparts as long as no disadvantage is encountered. We take the proposed mechanism of lateral gene transfer, together with the extent of laterally acquired genes observed in *T. oceanica*, as additional evidence for mixotrophic potential in *T. oceanica*.

In low-iron conditions we observe the joint up-regulation of a reverse transcriptase and a CRE-like recombinase, thereby providing an appropriate mechanistic basis for genome rearrangements via transposon mobilization. Moreover, these are activated at a time where enhanced DNA input through increased uptake of organic matter might be expected under natural conditions. Stress-induced transposon activation has been reported for higher organisms as well - for example, [46].

The observed plasticity of diatom genomes clearly has ecological implications, as bacterial inventions such as genes that are beneficial in a competitive context might quickly find their way into diatom species and strains. We therefore state that the hypothesized close integration of diatoms within the microbial loop (due to mixotrophy) and their remarkable genomic plasticity (as seen from lateral gene transfer) are keys to diatoms' ecological success: while mixotrophy opens up complex sources for carbon, energy and nutrients, the high capacity for lateral acquisition of genetic material facilitates adaptation in the context of the resulting competition with bacteria for organic matter, nutrients and iron.

### Iron uptake, cellular iron requirements and adaptation of species to low iron

For a better characterization of the complex cellular low-iron response it is necessary to distinguish the iron-specific aspects directed at counteracting the shortage of the limiting nutrient (substitution of iron proteins, induction of high-affinity iron uptake) from the rather general stress response directed at the situation of impaired growth and cellular starvation (cellular retrenchment, chlorosis, metabolic shift). With regard to the cellular iron economy we can define some constraints for beneficial adaptations to low-iron conditions [26].

Each evolutionary innovation that lowers the cellular iron requirements will favor survival under iron-limited conditions. Besides general biomass retrenchment - that is, the cellular adoption of a state with decreased biomass - the cell can actively lower its iron requirements through substitution of iron-containing proteins like ferredoxin or other metal enzymes that use iron as a cofactor. In addition to the well-known substitution pair ferredoxin/flavodoxin [10], we present evidence for the replacement of three metal-containing FBA enzymes by substitutes that use an amino acid-based Schiff-base catalysis instead. Differential regulation of diatom FBA genes was recently described for *P. tricornutum *and appears to be an evolutionarily conserved feature of several diatoms [27]. In *T. oceanica *we even find the apparently permanent functional replacement of an iron-rich counterpart as in the case of plastocyanin, though at the same time the genome harbors two genes encoding cytochrome c_6 _whose function and regulation remain unknown. Cytochrome c_6 _might be retained in the genome for replacing plastocyanin under specific circumstances like copper limitation, though this could not yet be observed [11]. Benefits may also arise from improved regulation of mutual substitution pairs - for example, as in the case of the transfer of the usually organellar ferredoxin gene *petF *to the nuclear compartment as found in *T. oceanica *[17].

Further, any improvement of the cellular iron-affinity and/or iron-uptake system will improve competitive fitness under low-iron conditions. The activation of a specific high-affinity uptake system as observed in yeast is expected to occur in diatoms as well. It has been found that reduction of Fe^3+ ^to Fe^2+ ^represents an essential step in uptake of organically complexed iron [47], suggesting that iron is extracted from its complexes prior to uptake. Accordingly, major players involved in iron uptake can be expected to be iron-complex-binding receptors, redox enzymes needed for extracting the iron from its complexes, possibly also specific iron-binding cell-surface molecules for short-term iron storage (like *D. salina *transferrins), ferric reductases and ferrous oxidases responsible for interconversion of the iron redox species +III and +II, and finally iron permeases for iron import. In this work on *T. oceanica *we were able to identify several candidate elements for the above groups. However, critical for iron-uptake kinetics is the overall iron-binding capacity of the cell surface, which directly depends on the sheer amount of iron-binding sites exposed to the cellular exterior [26]. A straightforward strategy to enhance the cellular capacity for iron binding is seen in the remarkable extent of domain duplications in iron-regulated cell-surface proteins. From the strong and exclusive expression in iron-limited conditions we speculate that ISIP1/ISIP3 are part of a specialized high-affinity iron-uptake system, with ISIP1 as the putative receptor (Figure 6). Carboxy-terminal endocytosis motifs as seen in ISIP1 (Figure 6) can also be found in other iron-regulated proteins (*T. oceanica *CREGx2, *P. tricornutum *ISIP2) and biochemical work is needed to confirm the location and proposed receptor function for these components. How far such features are species-specific adaptations or rather common to diatoms can only be clarified by a comparative genomic approach. Notably, screening for highly conserved iron-regulated genes in the genomes of *T. oceanica*, *T. pseudonana*, *P. tricornutum *and *F. cylindrus *revealed a correlation between the extent of a diatom's tolerance to low-iron and the presence of *ISIP1 *and *PETE*, which directly impact cellular iron economy and uptake.

## Conclusions

From their evolutionary roots diatoms already appear to be pre-adapted to low-iron conditions through the endosymbiotic acquisition of a 'red'-type photosynthetic machinery. While they have retained an implicit capacity for mixotrophy, the main contribution to cellular growth under conditions where iron or other nutrients limit the build-up of biomass, like in the iron-limited southern ocean or the oligotrophic Sargasso Sea, can be expected to stem from photosynthetic carbon assimilation.

The combined efforts in genomics, transcriptomics and proteomics reveal an unexpected metabolic flexibility in response to iron availability for *T. oceanica *CCMP1005. These responses include an extensive cellular retrenchment, the pronounced remodeling of bioenergetic pathways and an intrinsic shift to a mixotrophic life style. As a consequence of iron deprivation, the photosynthetic machinery undergoes remodeling into a photo-protected mode to cope with the overall decrease in photosynthetic electron transfer complexes. From the genomic and transcriptomic data we identify candidate components of a diatom high-affinity iron-uptake system, and we present a novel cellular strategy to enhance iron economy of phototrophic growth with the iron-regulated mutual substitution of three metal-containing FBA proteins.

The enormous genomic plasticity of *T. oceanica*, as seen from the large fraction of genes acquired through horizontal gene transfer, provides a platform for complex adaptations to the iron-limited ocean. The inferred dynamic exchange of genes between marine microbes suggests the genome of *T. oceanica *may not be an exceptional evolutionary invention, but rather that it may be seen as one possible outcome from a larger metagenomic gene pool. The future comprehensive characterization of this gene pool constitutes the ultimate challenge in appreciating the solutions that marine life found for defying the persistent shortage of iron in the open ocean.

## Materials and methods

### Strains, cultures and physiology

The sequenced strain *T. oceanica *(Hustedt) Hasle et Heimdal CCMP1005 [48] was obtained from the Center for Culture of Marine Phytoplankton [49].

*T. oceanica *cells were grown in 8 l batch cultures using iron-free f/2 nutrients in artificial seawater medium (ASW) [50] at 100 µE, 25°C and a 14/10 h light/dark cycle. Iron-replete cultures ('control') were supplied in excess of other essential nutrients (10 µM FeCl_3_); no iron was added to the iron-limited cultures ('stress'), except for residual iron from the ASW salts, promoting iron-limited growth. Cells were harvested at late exponential growth phase by filtration on 5 µm polycarbonate filters of 47 mm diameter, resuspended into a small volume of media, followed by centrifugation at 4°C for 10 minutes at 11,000 rpm. Cell pellets were frozen in liquid N_2 _and stored at -80°C.

For iron-limited cultures, iron-free techniques were applied as follows. Culture bottles were composed of plastic material, washed and incubated for some days with 1 N HCl and rinsed with ultrapure MilliQ water. All additional supply for iron-free work was washed with 1 N HCl and stored in closure bags until use. Iron-limited cultures were best achieved from a minimal inoculation volume of 10 to 20 µl or less than 10,000 cells.

Throughout this work we compare cells from late exponential growth phase, though we recommend to use iron-limited cells from late stationary phase when working on specifically low-iron responsive genes (for example, from compensation pairs or involved in iron uptake). For these, the expression level at the late stationary phase is found to be even higher than in the late exponential phase.

Total cellular protein was determined for iron-replete and iron-limited cells as follows. First, 85.5 million cells each were concentrated by centrifugation. The resulting pellets were frozen in liquid N_2 _and stored at -80°C. For protein determination, pellets were re-dissolved and lysed in 200 to 300 µl SDS/CO_3 _buffer with additional application of ultrasonication. Cell debris was precipitated by 4 minutes of centrifugation at room temperature and maximum rpm; 5 to 10 µl of the supernatant served as input for bicinchoninic acid (BCA) protein assay (Thermo Fisher Scientific, Waltham, MA, USA). SDS/CO_3 _buffer was composed of 4% SDS, 68 mM Na_2_CO_3 _and 0.4 mM phenylmethylsulfonyl fluoride (the latter was dissolved in EtOH to obtain a 100 mM stock solution).

The variable chlorophyll fluorescence F_v_/F_m _of *T. oceanica *cells was measured from fresh cultures with a PhytoPAM (PHYTO-PAM Phytoplankton Analyzer, Heinz Walz GMBH, Effeltrich, Germany) [51] upon 5 minutes of dark incubation.

Comparative genomics was done with genome data available at the Joint Genome Institute for *T. pseudonana *(Hustedt) Hasle & Heimdal CCMP1335 [4], *P. tricornutum *Bohlin CCAP1055/1 [5] and *F. cylindrus *(Grunow) Krieger CCMP1102 [19].

### Microscopy and confocal microscopy

*T. oceanica *was imaged *in vivo *using confocal laser scanning microscopy LSM 510 (Zeiss). Chlorophyll autofluorescence was excited at 488 nm (1% laser intensity), and emission recorded with a long pass (LP) 650 nm filter. Images were made using a Plan-Neofluar 40×1.3 oil objective (Zeiss). Z-section image series were captured with LSM 510 v3.2 software (Zeiss). Three-dimensional reconstructions of the chlorophyll fluorescence signal were made using the cell surface area-/cell volume-analyzing 'Surpass' program module in Imaris 7.1.1 (Bitplane, Zürich, Switzerland). Images were segmented using consistent threshold values. Surface area grain size was set at 0.1 µm. In 20 cells from both iron-replete and iron-limited cultures, cell dimensions were calculated from transmission image measurements based on a cylinder model. Chloroplast dimensions were calculated from three-dimensional chlorophyll autofluorescence signal reconstructions.

Despite imaging in a narrow time window (12:00 ± 2 h), growth in the *T. oceanica *cultures was not synchronized. Thus, the observed values for the parameters with a growth-dependent variability are accordingly distributed over a characteristic growth range. The representative cellular and chloroplast dimensions provided in Table 2 were therefore determined from the statistical mean of 20 cells by calculating back to a cell at the beginning of its cell cycle. For this purpose we used a regular cylinder that expands through a gradual two-fold increase of its height as a model for the diatom cell. Around the statistical mean from all cells (of a variable parameter like cell volume) we created a range whose higher end differs from the lower end by a factor of 2, thereby representing a two-fold increase of that parameter during growth of the diatom cell. The lower end of that range is given in Table 2 as a representative value for a 'cellular unit', that is, a freshly divided cell at the beginning of its cell cycle.

### Nucleic acid extraction and sequencing

*T. oceanica *CCMP1005 was grown as axenic clonal culture from a single cell isolate obtained from serial dilutions of a stock culture to extinction. Nuclear gDNA for sequencing of the *T. oceanica *genome was extracted from nutrient-replete cells and separated from organellar gDNA in a CsCl gradient (Figure 1) using the alternative cetyltrimethlyammonium bromide protocol for algae [52].The quality of nucleic acids was assessed from NanoDrop UV absorption profiles and agarose gel electrophoresis. Second generation sequencing technology was applied to the gDNA as follows. After mechanical shearing by nebulization, followed by end-repair, specific sequencing adaptors were ligated. The genomic DNA fragments were shotgun sequenced using massive parallel pyrosequencing [53] on a Roche 454 GS-FLX instrument (Roche, Penzberg, Germany) according to the manufacturer's protocol.

Total RNA for transcriptome sequencing was extracted from frozen pellets of iron-replete and iron-limited *T. oceanica *cells from late exponential growth phase using the QIAGEN (Hilden, Germany) RNeasy kit. RNA quality was assessed from NanoDrop UV absorption profiles and agarose gel electrophoresis. For reverse transcription of total RNA the SMART cDNA synthesis kit from Clontech (Mountain View, CA, USA) was used with 1 µg input material and 15 rounds of amplification. The size distribution of the obtained cDNA libraries were controlled with agarose gel electrophoresis and then subjected to Roche 454 sequencing as described above for gDNA.

### Transcriptomics

Global gene expression was assessed through Roche 454 massive parallel pyrosequencing of cDNA libraries prepared from total RNA extracted from iron-limited and iron-replete cultures. The 2× 95,000 sequence reads from both libraries were pooled, cleaned from adapter ends and processed in a combined assembly revealing 11,264 contigs (that is, transcript fragments) that map to approximately 6,500 distinct AUGUSTUS gene models (Additional file 10). The differential read contribution from the Fe(-) and Fe(+) libraries to each contig is taken as a measure for the differential transcription of the respective gene. For the purpose of statistically evaluating the gene expression level across our two cDNA libraries, we applied a log-likelihood ratio test statistic as described in Stekel *et al*. [22].

Differentially regulated genes were first screened with T-ACE [54], a transcriptome database browser that plots the assembled transcript fragments according to their differential regulation (Figure 4a) and provides information from BLAST analyses against the NCBI nr protein (NR) database and the Conserved Domain Database.

Models from selected genes were manually curated and annotated for protein function and location as described above (Additional file 6).

### RT-qPCR

RT-qPCR was done as in [17] and is described in the Supplementary Methods in Additional file 3. Primers are listed in Additional file 11. The differential regulation between high and low iron conditions with respect to 18S and RPB1 (threshold level) is shown in a ΔΔC_T _plot as calculated below:

$$\Delta \Delta {\text{C}}_{\text{T}}=\left[\Delta {\text{C}}_{\text{T}}@\text{Fe( + ) - }\Delta {\text{C}}_{\text{T}}@\text{Fe( - )}\right]$$

where ΔC_T = _(C_T _gene 1 - C_T _gene 2) and represents the difference between the qPCR threshold cycle values (C_T_) of gene 1 (the gene of interest) and gene 2 ( the house-keeping gene, either the 18S rRNA or RPB1).

(Figure S8 in Additional file 3).

### Proteomics

The proteomes of iron-replete and iron-limited cells were analyzed in a mass spectrometry approach. Differentially labeled iron-sufficient and iron-deficient cells were mixed at equal protein concentration and fractionated by SDS-PAGE. Protein bands were excised, digested in-gel with trypsin and subjected to LC-MS/MS analyses. Identification and quantification of peptides and proteins was performed using Proteomatic data evaluation pipelines [55] as follows. To provide candidate peptides in the database search step using OMSSA [56], several sequence sources were used: (1) the AUGUSTUS gene models, (2) Genomic Peptide Finder peptides [57], (3) high quality chloroplast protein models, (4) a set of manually curated protein models. Resulting peptide/spectral matches were filtered with a hit distinctiveness filter, using a threshold of 2. Peptide/spectral matches were further filtered with a dynamically determined E-value threshold to achieve an estimated false discovery rate of 1% [58]. Finally, all peptide/spectral matches with a precursor mass deviation >5 ppm were discarded.

A total of 1,695 peptides could be identified from two independent biological experiments and assigned to 767 protein groups. All identified peptides were subsequently quantified using qTrace [59], resulting in the quantification of 633 protein groups and an additional set of 88 quantified peptides, which were identified exclusively via Genomic Peptide Finder. For the determination of Fe(-)/Fe(+) protein ratios, all resulting combinations of peptide, SDS-PAGE band and charge state were grouped and all group ratios were combined into a total protein group ratio by calculating the median and interquartile range.

Data for differential protein expression as revealed from the mass spectrometry approach refers to equal amounts of total protein. For relating the observed regulation to a 'cellular unit', the ratio of cellular protein biomass [Fe(-)/Fe(+)] as determined from a BCA (bicinchoninic acid) protein assay needs to be taken into account.

### Bioinformatics

Bioinformatic analyses are described in the Supplementary Methods in Additional file 3.

### Data access

The *T. oceanica *CCMP1005 whole genome shotgun assembly is registered as bioproject 36595, the *T. oceanica *CCMP1005 transcriptome shotgun assembly is registered as bioproject 73029. The genomic and transcriptomic Roche 454 GS FLX sequence reads from this study have been submitted to the NCBI Sequence Read Archive [60] under accession numbers SRA045826 and SRA045825, respectively. The whole genome shotgun project has been deposited at DDBJ/EMBL/GenBank under the accession AGNL00000000. The version described in this paper is the first version, AGNL01000000. AUGUSTUS gene models deduced from the genome assembly have been assigned the gene loci accession numbers THAOC_00001 to THAOC_37921. The transcriptome assembly has been submitted to the NCBI Transcriptome Shotgun Assembly database [61] under accession numbers JP288099 to JP297710. The proteomics mass spectrometry mzML data associated with this manuscript may be downloaded from the Tranche repository [62] using the following hash: 'T9vKohxPxffmhOBsgb9kTlBKCrQIQYziH8hdonm9scou13EAFv57Uo+XYTj4d8XHbLRxR03+6WeDRSp2yhpp348wzWsAAAAAAABjUg=='. The data from this study can be accessed in an integrated form with the *Thalassiosira oceanica *Genome Browser [18].

## Abbreviations

CCAP: Culture Collection of Algae and Protozoa; CCMP: Culture Collection of Marine Phytoplankton; EST: expressed sequence tag; FBA: fructose-bisphosphate aldolase; FCP: fucoxanthin-chlorophyll a/c-binding protein; Fe(-): iron deplete; Fe(+): iron replete; gDNA: genomic DNA; HNLC: high-nitrate low-chlorophyll; HSF: heat shock factor; ISIP: iron starvation induced protein; LC-MS/MS: liquid chromatography-tandem mass spectrometry; LDLR: low-density lipoprotein receptor; LGT: lateral gene transfer; LHCSR: light harvesting complex stress responsive subunit; NCBI: National Center for Biotechnology Information; NRPS: non-ribosomal peptide synthase; ORF: open reading frame; PCR: polymerase chain reaction; PS: photosystem; RT-qPCR: reverse transcription-quantitative PCR.

## Competing interests

The authors declare that they have no competing interests.

## Authors' contributions

ML prepared the *T. oceanica *RNA used for transcriptomics, was involved in genomics, transcriptomics and proteomics data analysis, performed manual gene modeling and annotation, contributed the comparative genomics section, prepared the figures, and drafted the manuscript. MS developed the bioinformatic tools for analysis of the proteomics data and prepared Figure 4b. ML and MS conducted the gene prediction on the *T. oceanica *genome assembly using AUGUSTUS. ASR cultured the algae, carried out the RT-qPCR work and commented on the manuscript. LK performed the assembly of the Roche 454 reads and a general blast and domain annotation. RA set up the web-server and Generic Model Organism Database, annotated gene models, and helped with genome and proteome analysis. MAG carried out the confocal microscopy and the three-dimensional rendering of chlorophyll autofluorescence signals. JW and SVB carried out the proteomics experiment and participated in the proteomics analysis. MBS prepared the gDNA libraries, performed the Roche 454 sequencing and generated the initial assemblies. UCK prepared the cDNA libraries for transcriptomics. RGB and RA contributed the phylogenetic analysis of LGT genes. PR coordinated the sequencing and contributed to manuscript writing. MH coordinated the proteomics and contributed to manuscript writing. JLR coordinated the study, isolated the *T. oceanica *gDNA and contributed to manuscript writing. All authors read and approved the final manuscript.

## Supplementary Material

Additional file 1 **Supplemental table - BLAST analysis of AUGUSTUS-predicted protein models versus the NCBI Non-redundant Protein (nr) database and the Conserved Domain Database**. The table lists the best BLAST hits from a BLASTP analysis of AUGUSTUS-predicted protein models against NCBI nr protein and Conserved Domain Database. The file is in .xls format (compressed to .zip). Click here for file

Additional file 2 **Phylogenetic trees for *T. oceanica *LGT candidate genes**. The file comprises 254 unrooted, Newick-formatted trees containing candidate LGT genes as described in the main manuscript. Trees and corresponding support values were generated using FastTree. Novel *T. oceanica *genes are identified in the trees by a reference number followed by '_TO'. Homologs from the 'nr' database identified using BLAST are given in the form 'Genus_GI', except for matches to unnamed genera, which are shown as RefSeq GI number followed by 'X'. Visual inspection of trees was performed by importing this file into FigTree (Andrew Rambaut 2007) and assigning a root as described in the Supplementary Methods in Additional file 3. The file is in .tre format (compressed to .gz). Click here for file

Additional file 3 **Supplementary Methods and Figures**. The file provides a description of the RT-qPCR as well as of the bioinformatics methods (genome assembly and analysis, setup of the *T. oceanica *genome browser and phylogenetic analyses). It further contains supplementary Figures S1 to S9. Figure S1: taxonomy of genes acquired by lateral gene transfer (2). An overview of the taxonomic assignments for LGT candidate genes as revealed from a refined phylogenetic analysis. Figure S2: duplications of genes and domains occurring in genes whose regulation is dependent on the cellular supply with iron. Figure S3: metabolic shift. The change in relative abundance of organellar RNA between *T. oceanica *cells subjected to high or low iron. Figure S4: correlation plot P versus T. The extent of correlation between proteomics (P) and transcriptomics (T) data. Figure S5: FCP proteomics. The variation in the response of different FCP light-harvesting proteins to iron limitation. Figure S6: phylogenetic tree of flavodoxin proteins. A neighbor-joining tree of diatom flavodoxin proteins. Figure S7: phylogenetic tree of FBA proteins. A neighbor-joining tree of diatom fructose bisphosphate aldolase proteins. Figure S8: qPCR ΔΔC_T_. The differential regulation of genes in response to low iron. Data represent the change in transcript level for iron-limited *T. oceanica *when compared to an iron-replete control. Figure S9: promoter motif. The conservation across diatom species of a motif found in the promoters of several iron-regulated genes. The file is in .pdf format. Click here for file

Additional file 4 **Gene abbreviations list**. The file lists gene abbreviations and gene product descriptions for all genes mentioned in this paper. The file is in .xls format. Click here for file

Additional file 5 **Supplemental sequences - curated protein models**. The file lists 436 manually curated protein sequences from nuclear genes (with custom identifiers x1 to x12 and p1 to p455) together with the 158 proteins encoded by the organellar genomes in FASTA format. Click here for file

Additional file 6 **Supplemental table - manual transcriptome annotation**. The table lists annotation information for all protein sequences provided in Additional file 5. The file is in .xls format. Click here for file

Additional file 7 **Supplemental sequences - selected protein models discussed in the manuscript**. The file lists sequences of proteins that we explicitly refer to in the discussion of the low-iron response. The sequences are provided in FASTA format. Click here for file

Additional file 8 **Supplemental table - overview of low-iron responsive genes**. The table contains more detailed annotation information for all protein sequences provided in Additional file 7. Deviation of a custom curated model from its respective official 't1'-AUGUSTUS prediction is indicated as 'mod'. The file is in .xls format. Click here for file

Additional file 9 **Supplemental table - comparative genomics of selected genes with a putative adaptive relevance**. The table contains all official database identifiers for the gene products listed in Table 3. The file is in .xls format. Click here for file

Additional file 10 **Supplemental table - BLAST mapping of *T. oceanica *transcript fragments versus NCBI databases and other diatom genomes**. The table provides a comprehensive overview for all 11,264 transcript fragments with read statistics, best BLASTX hits to NCBI nr and Conserved Domain Database, and additional best TBLASTX hits to the genomes of *T. pseudonana*, *P. tricornutum *and *F. cylindrus*, notably including worldwide web link-outs to the respective orthologous genes of these species. The file is in .xls format (compressed to .zip). Click here for file

Additional file 11 **Supplemental table - primers used for RT-qPCR**. The table lists the sequences of all primers used in the RT-qPCR experiments. The file is in .xls format. Click here for file
