# Supplementary material for: Genome and low-iron response of an oceanic diatom adapted to chronic iron limitation
Source: Genome Biol. 2012 Jul 26;13(7):R66. doi: 10.1186/gb-2012-13-7-r66 (PMC3491386; doi:10.1186/gb-2012-13-7-r66)
Supplement: Additional file 3 — Supplementary Methods and Figures. The file provides a description of the RT-qPCR as well as of the bioinformatics methods (genome assembly and analysis, setup of the T. oceanica genome browser and phylogenetic analyses). It further contains supplementary Figures S1 to S9. Figure S1: taxonomy of genes acquired by lateral gene transfer (2). An overview of the taxonomic assignments for LGT candidate genes as revealed from a refined phylogenetic analysis. Figure S2: duplications of genes and domains occurring in genes whose regulation is dependent on the cellular supply with iron. Figure S3: metabolic shift. The change in relative abundance of organellar RNA between T. oceanica cells subjected to high or low iron. Figure S4: correlation plot P versus T. The extent of correlation between proteomics (P) and transcriptomics (T) data. Figure S5: FCP proteomics. The variation in the response of different FCP light-harvesting proteins to iron limitation. Figure S6: phylogenetic tree of flavodoxin proteins. A neighbor-joining tree of diatom flavodoxin proteins. Figure S7: phylogenetic tree of FBA proteins. A neighbor-joining tree of diatom fructose bisphosphate aldolase proteins. Figure S8: qPCR ΔΔCT. The differential regulation of genes in response to low iron. Data represent the change in transcript level for iron-limited T. oceanica when compared to an iron-replete control. Figure S9: promoter motif. The conservation across diatom species of a motif found in the promoters of several iron-regulated genes. The file is in .pdf format. [file gb-2012-13-7-r66-S3.PDF]

Supplemental Methods

Genome assembly, gene modelling and genome analysis

The Roche 454 sequence reads obtained from genomic (g)DNA were pooled, cleaned from adapter ends and assembled with the newbler (Margulies et al. 2005) and the TGICL assembler (Pertea et al. 2003).

The gene prediction tool AUGUSTUS (Stanke et al. 2006) was trained with the public available genomes and gene models of *T. pseudonana*, *P. tricornutum* and *F. cylindrus* to obtain proper diatom-specific parameter settings for searching genes in the genome of *T. oceanica*. AUGUSTUS was run on the *T. oceanica* genome assembly using transcriptomic ESTs and mass spectrometry-based GPF peptides of *T. oceanica* (Specht et al. 2011) as well as best BLAST hits from a search of all available gene models (BLASTX) and genomes (TBLASTX) of the above diatoms against the *T. oceanica* genome assembly as additional user-defined constraints for improved gene prediction.

The AUGUSTUS gene models were subjected to a BLAST search against NCBI non-redundant (nr) protein database and NCBI Conserved Domain Database (CDD). The best BLASTP hits of protein models against NCBI nr were taken as preliminary automatic annotation.

For manual improvement of gene models and manual annotation we also made use of the GENSCAN webserver (Burge and Karlin 1997), InterproScan (Hunter et al. 2009) and diverse bioinformatics tools like TMHMM (transmembrane regions), SIGNALP (signal peptides or anchors) and TARGETP (targeting to cellular compartments) from the CBS (Center for Biological Sequence analysis) Prediction Servers at http://www.cbs.dtu.dk/services/.

Comparative genomics was done as follows. The conserved promoter motif was identified from pairwise comparisons of promoter sequences with dotlet (Junier and Pagni 2000) and MEME (Bailey and Elkan 1994). Characterization of the ISIP1 protein was supported by PSIPRED secondary structure prediction (Bryson et al. 2007), the curated annotation of the human LDL receptor (Jeon and Blacklow 2005) and the information on cystein patterns of metalloproteins found in the Metalloprotein Database and Browser (Castagnetto et al. 2000). Dotlet was also used for the identification of domain duplications.

All BLAST (Altschul et al. 2007) searches were done using the local BLAST package from NCBI. BioEdit (Hall 1999) was used for manual sequence manipulation and visual inspection of sequences and sequence alignments.

Genome browser

For the current *T. oceanica* CCMP1005 genome assembly a GMOD GBrowse version 2.26 (Stein et al. 2002) downloaded from http://www.gmod.org/ was set up on a stand-alone web-server accessible at http://bose.geomar.de/cgi-bin/gbrowse/Toceanica/. It is a highly customisable open-source tool suitable for displaying assembly and annotation information for genome sequencing projects. The second generation of given browser allows the user to configure each track separately (colours, shape, packing etc.) and change their order. The diverse sequence data available for *T. oceanica* was mapped to the genome and is presented as separate tracks, specifically: AUGUSTUS gene models including alternative predictions ("Genes", "CDS"), selected models for interpretation of the *T. oceanica* low-iron response ("Low-iron response models"), EST clusters ("ESTs"), proteomics peptides ("GPF"), and mapping of the complete proteomes from all stramenopile species sequenced to date ("Models") that are available through the JGI genome portal. AUGUSTUS gene models and ESTs were mapped with BLAT (Kent 2002) using default settings. Protein models from JGI were mapped to *T. oceanica* scaffolds using TBLASTN with an E-value cut-off 1.0E-03. All results were converted to GFF3 format (http://www.sequenceontology.org/gff3.shtml) and imported to the GBrowse database. Users can browse *T. oceanica* genome by searching specific regions from scaffolds, inserting protein name or other information present in the automatic annotation.

Phylogenetic analysis of genes acquired by lateral gene transfer

Candidate laterally transferred genes were subjected to phylogenetic analysis in order to confirm their mode of inheritance and identify putative donor or recipient lineages. Protein sequences of the 696 candidate genes were first compared to each other using BLASTP version 2.2.23. Sets of proteins that matched each other with an e-value of 10^{-5} or less were grouped into clusters. A total of 379 clusters resulted, with the largest containing 64 proteins and 261 clusters containing a single protein. Each cluster was then compared against the NCBI 'nr' database using BLASTP to identify putatively homologous sequences from other organisms. For each query protein, we identified the best match to the 'nr' database and the corresponding e-value, and included this and all other matches within 20 orders of magnitude. For example, if the best match of query sequence Q was to subject S with an e-value of 10^{-120}, then all hits to nr with e-values between 10^{-120} and 10^{-100} were included in Q's result set. In cases where the best e-value was truncated to zero, we included all results with an e-value of 10^{-150} or less. A global maximum e-value of 10^{-10} was applied for the screening of hits. The result sets for each query protein in a cluster were assembled into a single set of sequences representing putative homologs of that cluster. Result sets were further restricted by removing any 'nr' sequences that were less than 75% as long as the shortest *T. oceanica* sequence in a

cluster, or longer than the longest *T. oceanica* sequence by a similar margin. The resulting 379 homologous sets ranged in size from 1 (57 clusters) to 1618 proteins.

The 254 clusters with 4 or more proteins (including sequences from both *T. oceanica* and the 'nr' database) were subjected to phylogenetic analysis as follows. First, all sequences in a cluster were aligned using MUSCLE version 3.7 with the parameters '-maxiters 1 -diags -sv -distance1 kbit20_3'. This alignment was then used to build a hidden Markov model using HMMER 3.0 (Eddy 2011), and sequences were then realigned to this HMM in order to generate posterior probability information. Sequences were removed from the alignment if their average reported posterior score over all aligned residues was less than 5.0, and alignment columns were removed if their score failed to meet the same threshold. The three-step procedure of alignment construction with MUSCLE, HMM construction and HMM realignment was repeated until no further sequences were removed. Phylogenetic trees were inferred from completed alignments using FastTree 2.1.0 SSE3 (Price et al. 2010) with options '-gamma -spr 4 -mlacc 2 -slownni'.

To identify groups neighboring *T. oceanica* genes in the constructed trees, we assigned a root based on the recovered topology. Each lineage in an unrooted tree has two candidate sister lineages, we assumed the sister to be the lineage that had fewer leaves. In cases where both candidate sister groups had the same number of leaves, we assigned as sister the group that had the shortest total branch length between its leaves and the *T. oceanica* sequence. Taxonomic labels at different ranks were assigned by querying the NCBI Taxonomy database with the gi numbers of interest. Sequences whose identified sister lineages contained only stramenopile taxa were excluded from the list of candidate LGT genes, and paralogous sets of genes from *T. oceanica* were only counted once.

RT-qPCR

RT-qPCR was performed as described in (Lommer et al. 2010):

Based on the predicted transcript sequences derived from the genome assembly data, sets of primers were designed and optimized to detect gene specific amplicons of approx. 100 bp with uniformly high amplification efficiency (> 95 %). A local BLASTN analysis of the primers against all sequences available for *T. oceanica* confirmed the specificity of the primers for their respective genes.

cDNA template was prepared from 1µg RNA by reverse transcription using the QuantiTect Rev. Transcription Kit (QIAGEN), followed by digestion of residual DNA using the included gDNA wipeout reagents. The cDNA was diluted to 0.5 ng μl^{-1} and 2.5 ng were used per qPCR reaction run on an ABI Prism 7000 (Applied Biosystems). Cycling conditions were 2 min at 50°C (once), 2 min at 95°C (once), and 40 cycles of 95°C for 0:15 min, followed by

0:30 min at 60°C. The qPCR mixtures contained 12.5 µl SYBR qPCR SUPERMIX W/ROX (Invitrogen), 0.5 µl of 10 µM forward and reverse primer each, 6.5 µl H_2O and 5 µl of cDNA template.

Gene expression was assessed as the mean from the C_T values of 2-4 replicate reactions at a threshold level of 0.2. Relative expression of genes with respect to the 18S-rRNA gene was calculated using the ΔC_T method ($\Delta C_T[geneX] = C_T[geneX] - C_T[18S]$) as mean ± s.e.m. from three biological replicates. The differential expression between iron-replete and iron-deplete conditions was visualized as $\Delta\Delta C_T$, with $\Delta\Delta C_T[geneX] = (\Delta C_T[geneX]^{Fe+}) - (\Delta C_T[geneX]^{Fe-})$.

Phylogenetic trees for diatom fllavodoxin and FBA proteins

For construction of phylogenetic trees protein sequences were aligned with CLUSTAL W v1.83 (Thompson et al. 1994). Resulting alignments were converted to Phylip 4 format. Neighbour joining trees were constructed with TREECON v1.3b (Van de Peer et al. 1994) using midpoint rooting, so that clusters of orthologous sequences were separated best possible and most branches showed similar length, thereby reflecting an even evolutionary progression for most proteins.

References for Supplemental Methods

- Altschul SF, Gish W, Miller W, Myers EW, Lipman DJ. 1990. Basic Local Alignment Search Tool. *J Mol Biol* **215**: 403-410.
- Bailey TL, Elkan C. 1994. Fitting a mixture model by expectation maximization to discover motifs in biopolymers. In *Proceedings of the Second International Conference on Intelligent Systems for Molecular Biology* (ed. R Altman, D Brutlag, P Karp, R Lathrop, D Searls), pp. 28-36. AAAI Press, Menlo Park, California.
- Bryson K, McGuffin LJ, Marsden RL, Ward JJ, Sodhi JS, Jones DT. 2005. Protein structure prediction servers at University College London. *Nucl Acids Res* **33**: W36-W38.
- Burge C, Karlin S. 1997. Prediction of complete gene structures in human genomic DNA. *J Mol Biol* **268**: 78-94.
- Castagnetto JM, Hennessy SW, Roberts VA, Getzoff ED, Tainer JA, Pique ME. 2002. MDB: the Metalloprotein Database and Browser at The Scripps Research Institute. *Nucleic Acids Res* **30**: 379-382.
- Eddy SR. 2011. Accelerated profile HMM searches. *PLoS Comput Biol* **7**:e1002195.
- Edgar RC. 2004. MUSCLE: multiple sequence alignment with high accuracy and high throughput. *Nucleic Acids Res* **32**:1792-1797.
- Hall TA. 1999. BioEdit: a user-friendly biological sequence alignment editor and analysis program for Windows 95/98/NT. *Nucleic Acids Symp Ser* **41**: 95-98.

- Hunter S, Apweiler R, Attwood TK, Bairoch A, Bateman A, Binns D, Bork P, Das U, Daugherty L, Duquenne L, et al. 2009. InterPro: the integrative protein signature database. *Nucleic Acids Res* **37**: D211-D215.
- Jeon H, Blacklow SC. 2005. Structure and physiologic function of the low-density lipoprotein receptor. *Annu Rev Biochem* **74**: 535-562.
- Junier T, Pagni M. 2000. Dotlet: diagonal plots in a web browser. *Bioinformatics* **16**: 178-179.
- Kent WJ. 2002. BLAT - the BLAST-like alignment tool. *Genome Res* **12**: 656-664.
- Lommer M, Roy AS, Schilhabel M, Schreiber S, Rosenstiel P, LaRoche J. 2010. Recent transfer of an iron-regulated gene from the plastid to the nuclear genome in an oceanic diatom adapted to chronic iron limitation. *BMC Genomics* **11**: 718. DOI: 10.1186/1471-2164-11-718.
- Margulies M, Egholm M, Altman WE, Attiya S, Bader JS, Bemben LA, Berka J, Braverman MS, Chen YJ, Chen Z, et al. 2005. Genome sequencing in microfabricated high-density picolitre reactors. *Nature* **437**: 376-380.
- Pertea G, Huang X, Liang F, Antonescu V, Sultana R, Karamycheva S, Lee Y, White J, Cheung F, Parvizi B, et al. 2003. TIGR Gene Indices clustering tools (TGICL): A software system for fast clustering of large EST datasets. *Bioinformatics* **19**: 651–652.
- Price MN, Dehal PS, Arkin AP. 2010. FastTree 2 - approximately maximum-likelihood trees for large alignments. *PLoS One* **5**:e9490.
- Specht M, Stanke M, Terashima M, Naumann-Busch B, Janßen I, Höhner R, Hom EFY, Liang C, Hippler M. 2011. Concerted action of the new Genomic Peptide Finder and AUGUSTUS allows for automated proteogenomic annotation of the Chlamydomonas reinhardtii genome. *Proteomics* **11**: 1814-1823.
- Stanke M, Tzvetkova A, Morgenstern B. 2006. AUGUSTUS at EGASP: using EST, protein and genomic alignments for improved gene prediction in the human genome. *Genome Biology* **7**: S11. DOI: 10.1186/gb-2006-7-s1-s11.
- Stein LD, Mungall C, Shu S, Caudy M, Mangone M, Day A, Nickerson E, Stajich JE, Harris TW, Arva A, et al. 2002. The generic genome browser: a building block for a model organism system database. *Genome Res* **12**: 1599-1610.
- Thompson JD, Higgins DG, Gibson TJ. 1994. CLUSTAL W: improving the sensitivity of progressive multiple sequence alignment through sequence weighting, position-specific gap penalties and weight matrix choice. *Nucl Acids Res* **22**:4673-4680.

- Van de Peer Y, De Wachter R. 1994. TREECON for Windows: a software package for the construction and drawing of evolutionary trees for the Microsoft Windows environment. *Comput Appl Biosci* **10**:569-570.

Supplemental Figures

Fig. S1 - Taxonomy of genes acquired by lateral gene transfer

A total of 530 genes were suggested to be derived from lateral gene transfer events from a screening of their best BLAST hits. A subset of 200 genes for which a sufficient number of orthologs could be recruited was subjected to a refined phylogenetic analysis to test for the postulated lateral origin. The inferred taxonomic distribution at superkingdom (top) and at phylum level (bottom) is comparable to the one obtained from best BLAST hit screening shown in Figure 2, with approximately 55% of the genes originating from eukaryotic ancestors . The occasional heterogenous mix of taxa in sister lineages as represented by "*Multiple* Superkingdoms/Eukaryota/Bacteria" categories suggests frequent gene transfer between major groups.

Fig. S2 - Duplications of Iron-Regulated Genes and their Domains in *Thalassiosira oceanica*

Several genes involved in the low-iron response of *T. oceanica* like *ISIP1*, *FLDA* or *CREG* are conserved across diatom species and are also present in *P. tricornutum*. While in *P. tricornutum* these are single-copy genes encoding for single-domain proteins (bottom), the situation in *T. oceanica* is different (top). Here we find additional paralogous versions of these genes (*ISIP1*, *FLDA*), as well as diverse examples of domain duplications (*CREGx2*, *CYTC6B*, *ISIP2x8*).

Paralogous genes in *T. oceanica* are independently regulated, with only one of the *ISIP1* and *flavodoxin* genes being responsive to low-iron. Differential expression of *T. oceanica* genes with respect to iron availability is indicated by EST support at low-iron vs high-iron.

Fig. S3 - Metabolic Shift

The transcript level from organellar rRNA operons is indicative of organellar translational activity. The observed relative abundance of organellar rRNA indicates an increased role of mitochondrial metabolism upon iron-limitation (metabolic shift). Accordingly, we find a pronounced decrease in chloroplast rRNA during the course of chloroplast retrenchment.

Fig. S4 - Correlation Between Gene Expression on Protein vs Transcript Level as Revealed from Proteomics and Transcriptomics Data

Proteomics and transcriptomics data were compared by plotting the differential gene expression ratios on protein versus transcript level as inferred from the respective approaches. Numbers and circle sizes indicate protein counts in every bin; bins are labelled with $\log_2$

ratios. The plot shows an increased dynamic range of transcriptomics ratios compared to proteomics ratios.

Fig. S5 - Differential Regulation of FCPs (Proteomics Data)

This plot shows the FCP subset of the median protein ratios plotted in Fig. 4B as determined by mass spectrometry, with error bars being defined by the first and third quartile of all observations for the respective gene product. In response to low-iron, fucoxanthin-chlorophyll a/c-binding proteins show differential regulation on the protein expression level, with two LI818-like proteins being up-regulated.

Fig. S6 - Phylogeny of Diatom Flavodoxin Proteins

The phylogenetic relationship between diatom flavodoxin proteins is shown as neighbour-joining tree (midpoint rooting). Flavodoxin proteins fall in two separate clades [34]. Most clade II flavodoxins are capable of replacing ferredoxin in the photosynthetic electron transport chain and are regulated by iron availability. Clade I flavodoxins are highly conserved between species and not responsive to low-iron. They are predicted to contain a signal peptide and may act in a functional context different from photosynthesis. Official NCBI accession numbers or JGI and AUGUSTUS identifiers are provided for each sequence.

Fig. S7 - Phylogeny of Diatom FBA Proteins

The phylogenetic relationship between diatom fructose-bisphosphate aldolase (FBA) proteins is shown as neighbour-joining tree (midpoint rooting). There are two classes of FBA proteins that differ in their catalytic mode. Class II FBAs act by metal catalysis and are highly conserved between species. Class I FBAs use a Schiff-base catalysis instead and are assumed to replace their class II counterparts under low-iron conditions in the three compartments chloroplast pyrenoid, chloroplast stroma and the cytosol [26]. Official NCBI accession numbers or JGI and AUGUSTUS identifiers are provided for each sequence.

Fig. S8 - RT-qPCR Assays Confirm the Iron-Dependent Regulation of Diverse Genes ($\Delta\Delta C_T$)

A subselection of genes was tested with RT-qPCR to confirm the transcriptomics-based evidence for their differential transcription under high and low iron. Notably the specificity of qPCR allows for discrimination between closely related paralogs like *ISIP1A* & *ISIP1B* or *FLDA1* & *FLDA2*.

The differential regulation between high and low iron conditions with respect to the standard genes 18S and RPB1 is visualized by a $\Delta\Delta C_T$ plot. The $\Delta\Delta C_T$ amplitude indicates the difference between relative transcript levels (normalized to 18S as ΔC_T), while the threshold

level represents the $\Delta\Delta C_T$ of the cell constant standard RPB1, the gene for the nuclear RNA polymerase II largest subunit. $\Delta\Delta C_T$ values higher than this threshold are truly upregulated under low-iron conditions. A strong increase in cellular transcript levels under iron limitation is observed for ISIP1A, ISIP3, FLDA1, cpRPL36 and FBA4, while the transcript level of PETF is decreased.

Fig. S9 - Conserved Palindromic Motif in the Promoter Region of Iron-Regulated Diatom Genes

We selected the *ISIP1*, *FLDA* and *FBA3* genes from each of the three diatoms *T. oceanica*, *P. tricornutum* and *F. cylindrus* for a comparative analysis of their respective promoter regions up to 300 bases upstream the translation start. A common conserved palindromic motif with the consensus “ACACGTGC” and located around pos. -200 from the respective translation starts was identified by the motif finder tool MEME (top). A motif alignment for the chosen genes (center) shows tolerance to some variation, though motif positions 3 (A) and 5 (G) are completely conserved in this small sample of promoters. Motif variants are also found in the promoters of less responsive genes like *T. oceanica ICL* or *NRPS2* (bottom), though at more distant promoter positions.

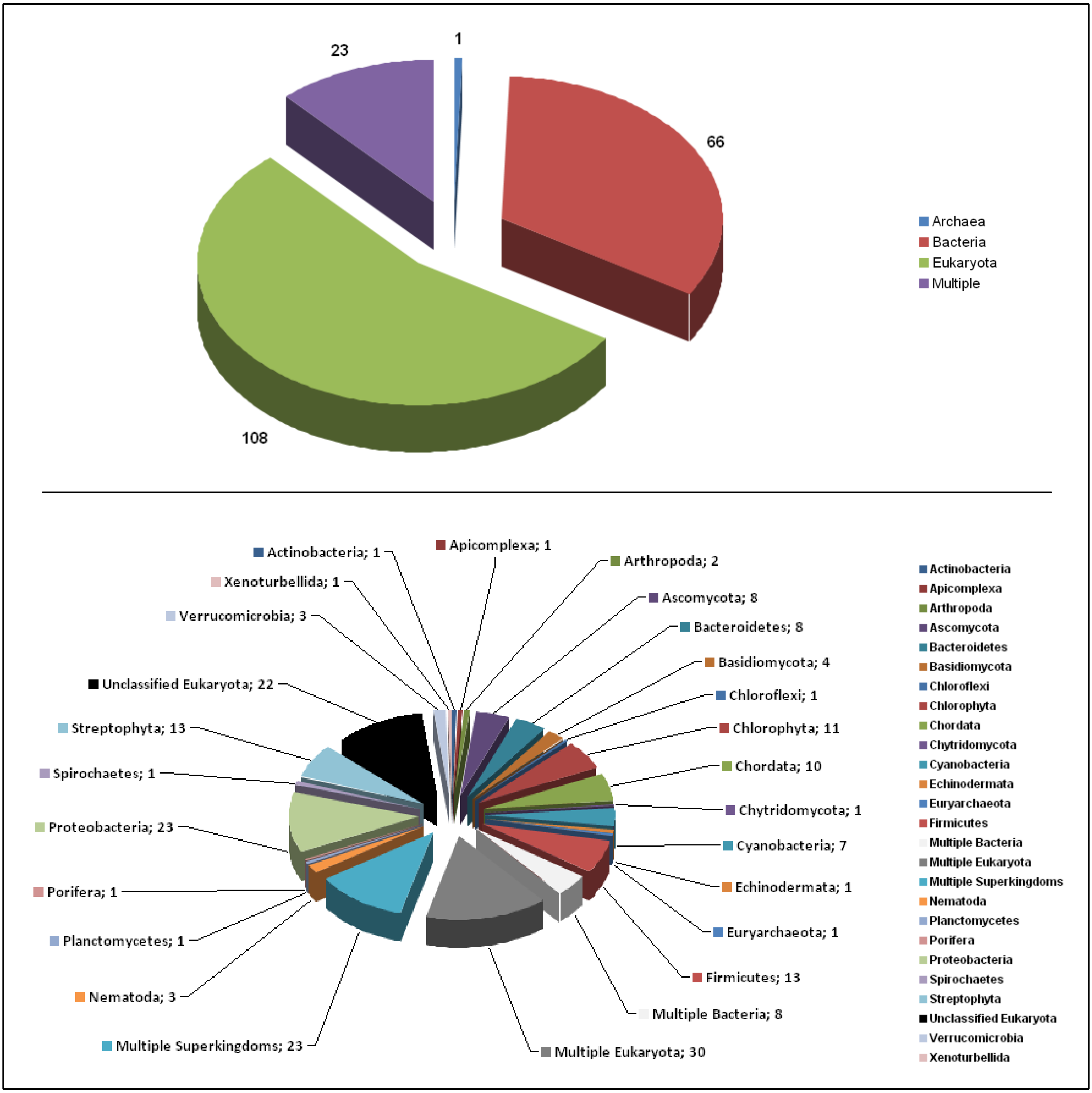

Figure S1: Taxonomy of genes acquired from lateral gene transfer

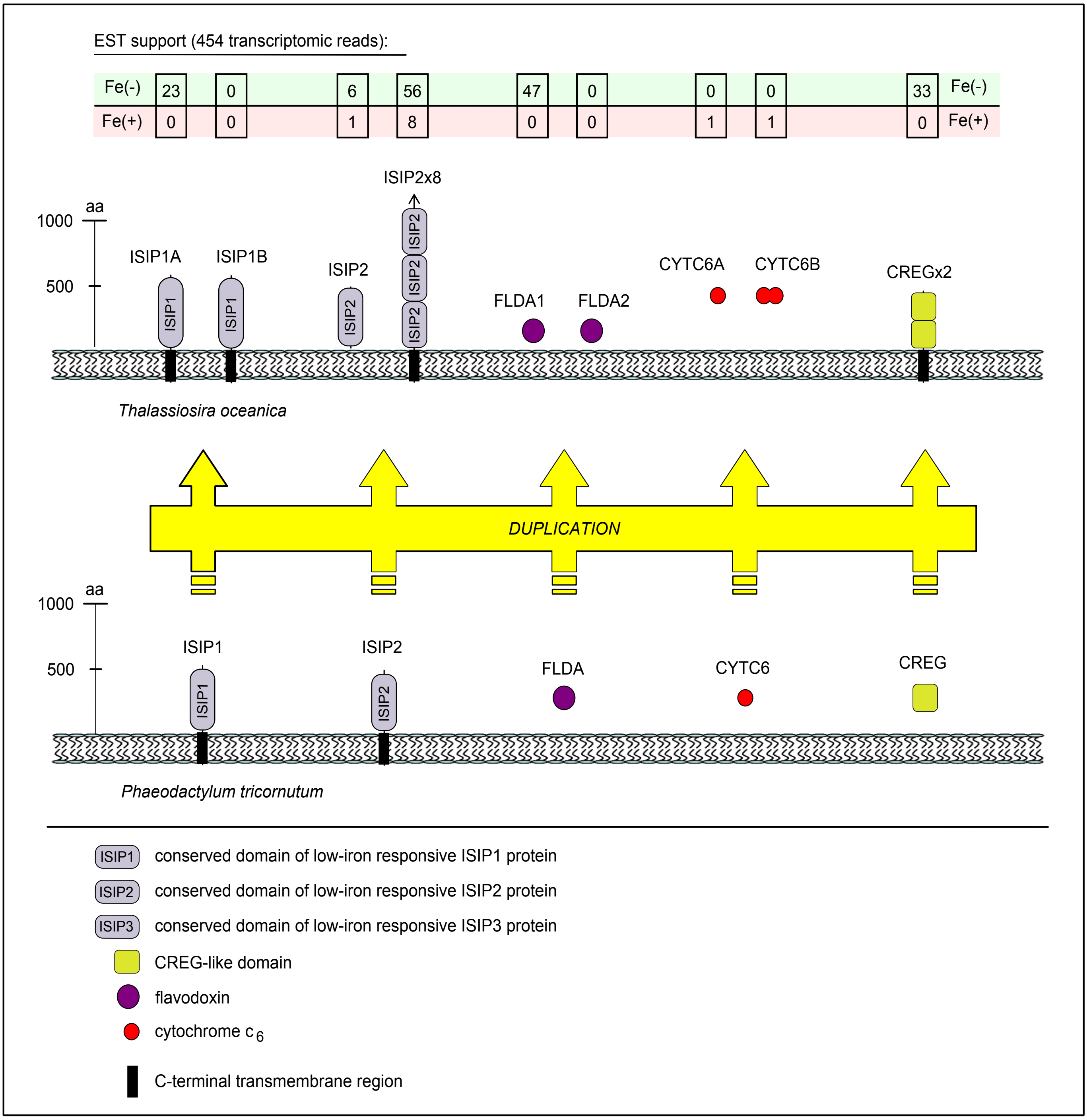

Figure S2: Duplications of iron-regulated genes and their domains in *Thalassiosira oceanica*

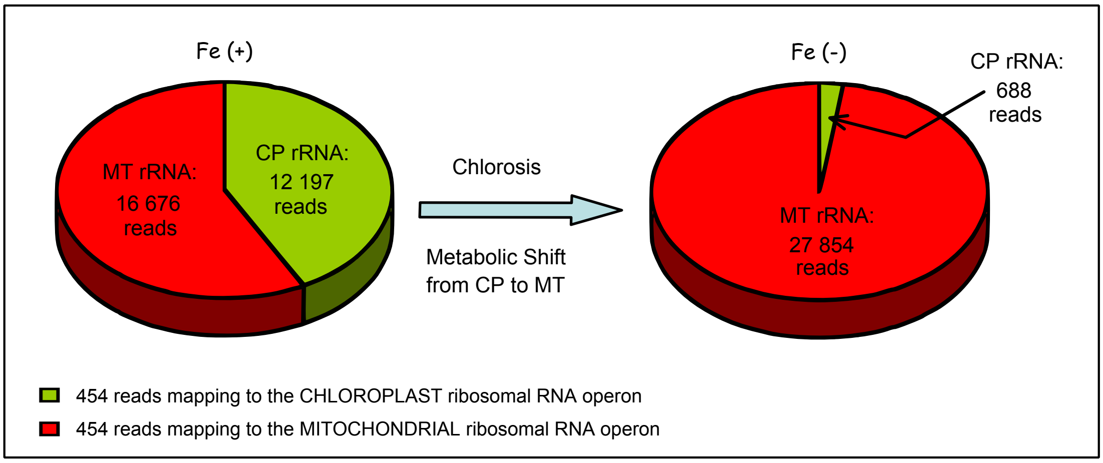

Figure S3: Metabolic shift

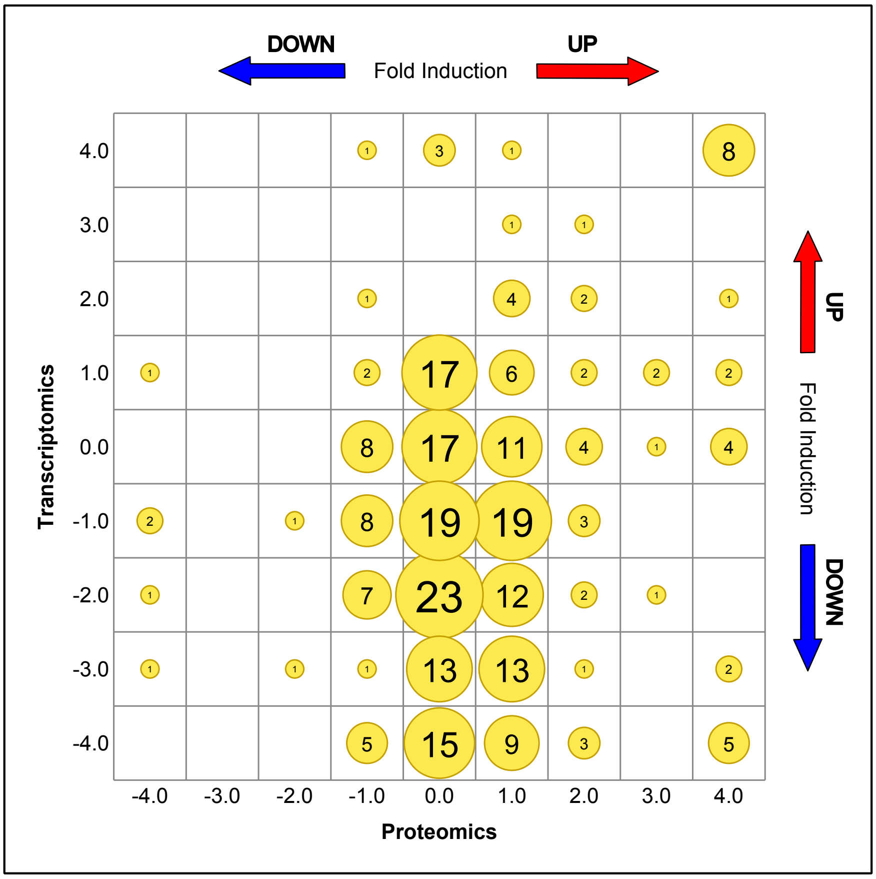

Figure S4: Correlation between gene expression on protein vs transcript level as revealed from proteomics and transcriptomics data

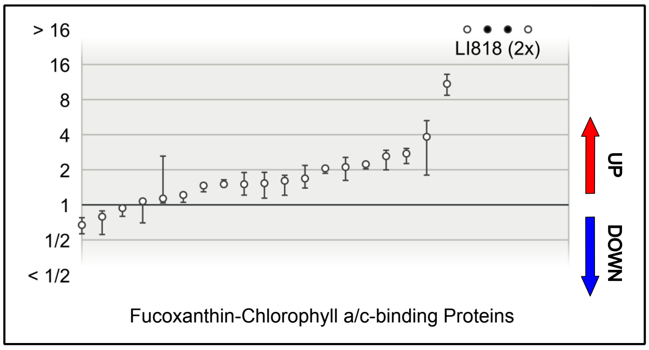

Figure S5: Differential regulation of FCPs (proteomics data)

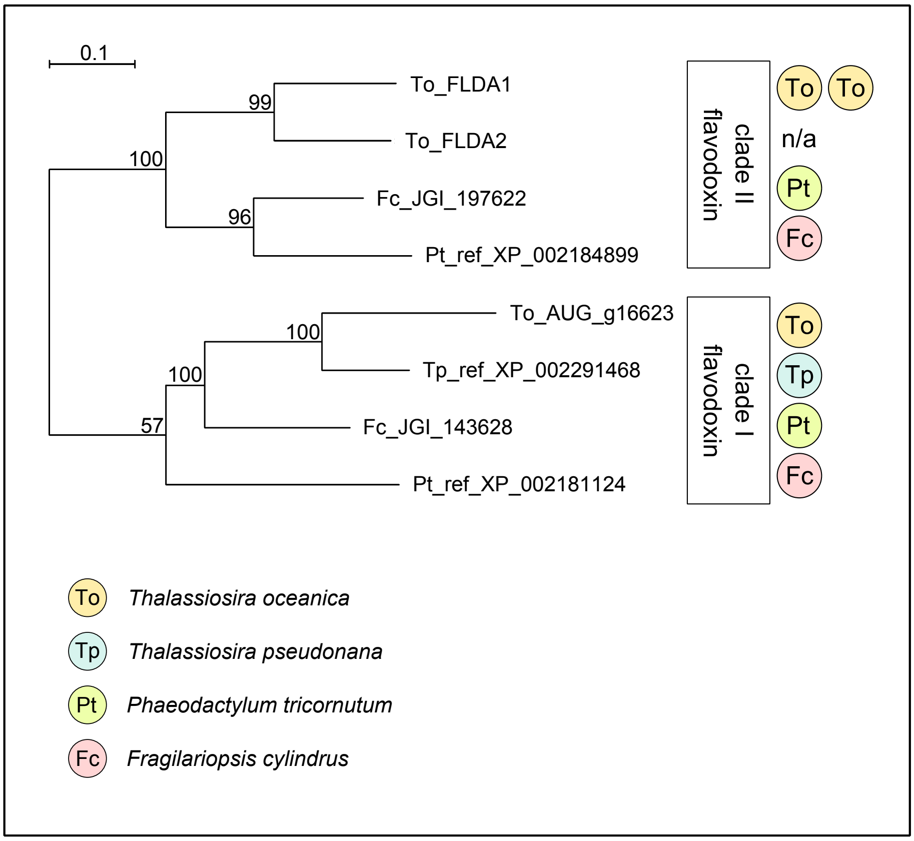

Figure S6: Phylogeny of diatom flavodoxin proteins

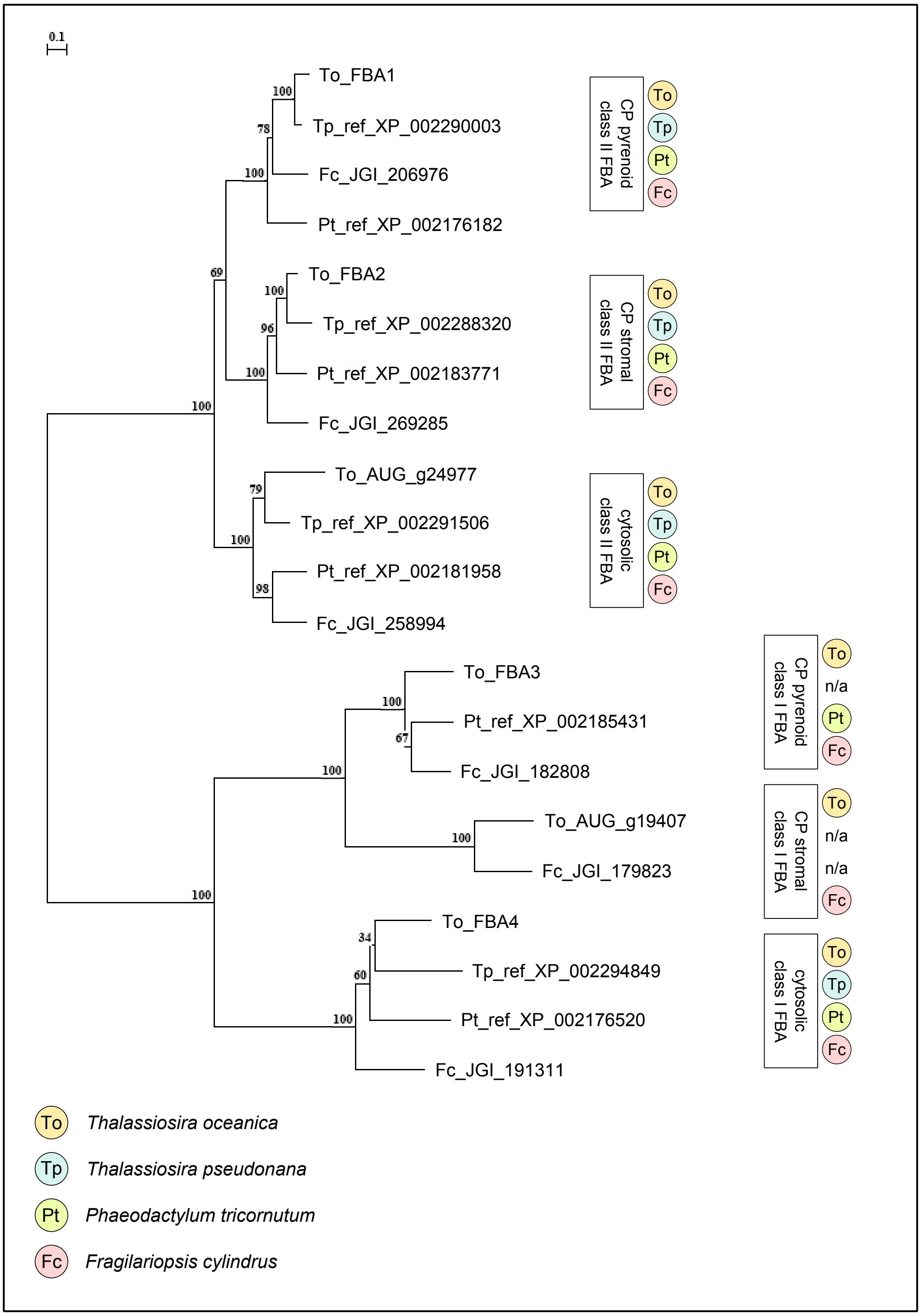

Figure S7: Phylogeny of diatom FBA proteins

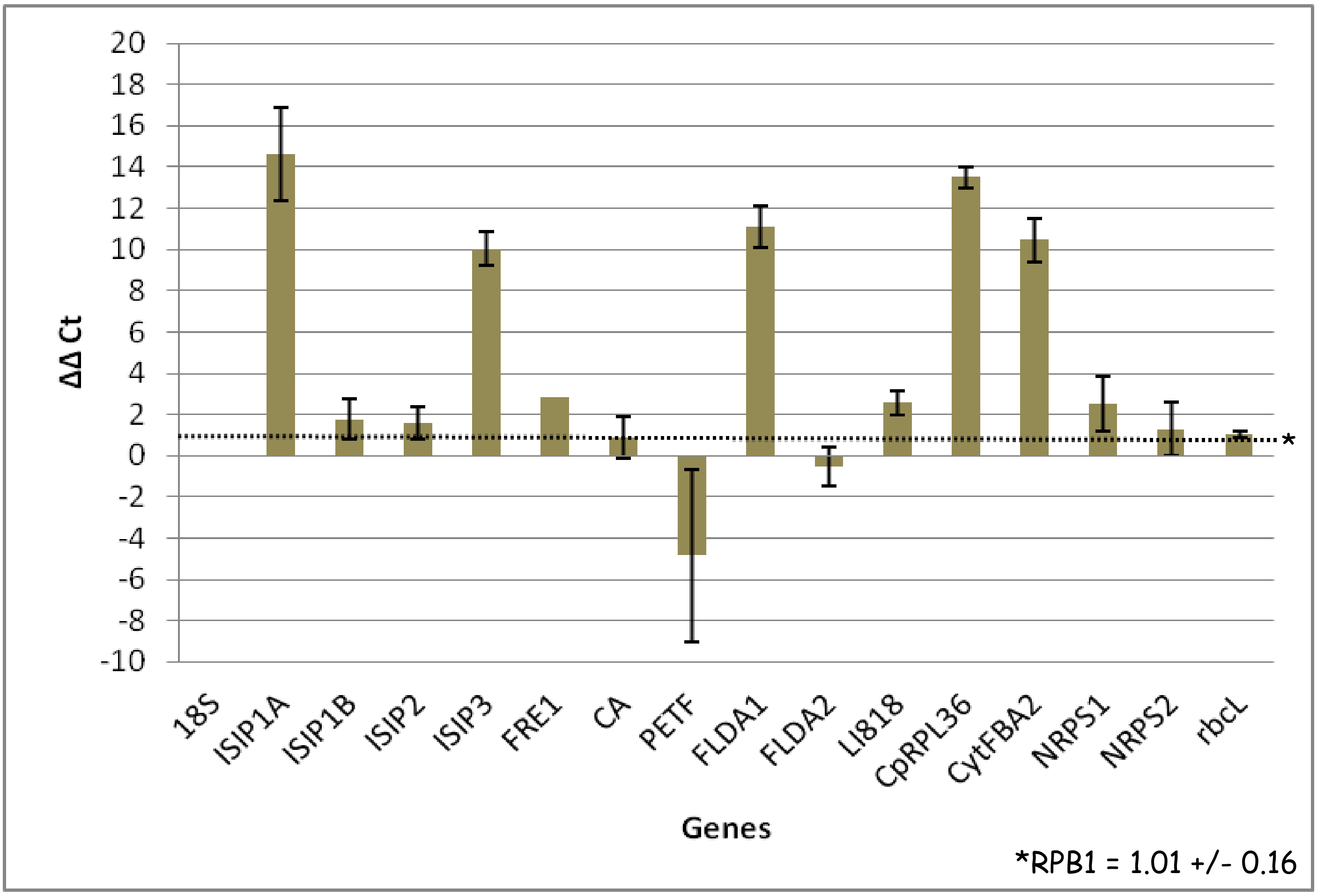

$$\Delta\Delta C_T^{\text{Gene X}} = (\Delta C_T^{\text{Gene X}})_{Fe+} - (\Delta C_T^{\text{Gene X}})_{Fe-}$$

ISIP1A	Iron Starvation Induced Protein 1	*FLDA1*	Flavodoxin
ISIP1B	Paralog of Iron Starvation Induced Protein 1	*FLDA2*	Paralog of Flavodoxin
ISIP2	Iron Starvation Induced Protein 2	*LI818*	LI818-type FCP
ISIP3	Iron Starvation Induced Protein 3	*cpRPL36*	Chloroplast Ribosomal Protein L36
FRE1	Ferric Reductase 1	*cytFBA2*	Cytosolic Class I Aldolase (FBA4)
CA	Carbonic Anhydrase	*NRPS1*	Non-Ribosomal Peptide Synthase 1
PET E	Plastocyanin	*NRPS2*	Non-Ribosomal Peptide Synthase 2
PET F	Ferredoxin	*rbcL*	Rubisco large subunit

Figure S8: RT-qPCR confirms the iron-dependent regulation of diverse genes ($\Delta\Delta C_T$)

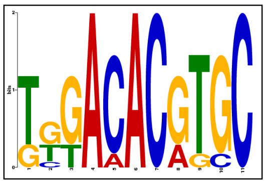

		-3	1 2 3 4 5 6 7 8	+3	pos. relative to translation start
T.o.	*ISIP1A*	GGG	ACACGTGC	CGC	- 218
	FLDA1	TGT	ACACGGGC	CAC	- 226
	FBA3	TTT	ACACGTGC	ACT	- 195
P.t.	*ISIP1*	TCA	GCACGTGT	CCA	- 136
	FLDA	TTG	ACACGTGC	CAA	- 172
	FBA3	TGG	ACACGTGC	CAA	- 218
F.c.	*ISIP1*	AGA	GAAAGTGC	AAC	- 244
	FLDA	GAC	GAACGTGC	GTT	- 201
	FBA3	GAC	ACACGTAC	GCG	- 241
T.o.	*FRE2*	GAG	ACACGTTC	CTA	- 183
	ICL	GAA	ACACGTGT	TCA	- 298
	NRPS2	CAG	ACACGGGC	ATC	- 376

consensus **ACACGTGC**

T.o. *Thalassiosira oceanica*
P.t. *Phaeodactylum tricornutum*
F.c. *Fragilariopsis cylindrus*

ISIP1(A) Iron Starvation Induced Protein 1
FLDA(1) chloroplast Flavodoxin
FBA3 chloroplast Fructose Bisphosphate Aldolase 2
FRE2 Ferric Reductase 2
ICL mitochondrial Isocitrate Lyase
NRPS2 Non-Ribosomal Peptide Synthase 2

Figure S9: Conserved palindromic motif in the promoter regions of iron-regulated diatom genes
